# The Exceptional Challenges of Si‐Based Anodes and the Eternal Hunt for Electrolyte Additives: A Perspective on the Example of a Novel Dioxazaborole‐Based Molecule

**DOI:** 10.1002/smsc.70429

**Published:** 2026-10-22

**Authors:** Anindityo Arifiadi, Steffen Heuvel, Lisa Balke, Niklas M. Abke, Alexandros Tsoufios, Julius Buchmann, Benjamin T. Manard, C. Derrick Quarles, Dominik Voigt, Sascha Nowak, Martin Winter, Uwe Karst, Frank Glorius, Johannes Kasnatscheew

**Affiliations:** ^1^ MEET Battery Research Center Institute of Physical Chemistry, University of Münster Münster Germany; ^2^ International Graduate School for Battery Chemistry, Characterization, Analysis, Recycling and Application (BACCARA) University of Münster Münster Germany; ^3^ Institute of Organic Chemistry, University of Münster Münster Germany; ^4^ Institute of Inorganic and Analytical Chemistry, University of Münster Münster Germany; ^5^ Chemical Sciences Division Oak Ridge National Laboratory Oak Ridge Tennessee USA; ^6^ Elemental Scientific, Inc. Omaha Nebraska USA; ^7^ Helmholtz Institute Münster, IMD‐4, Forschungszentrum Jülich GmbH Münster Germany

**Keywords:** anode, cathode, diffusion, electrode, electrolyte, materials science, passivation, rational design, redox, specific energy

## Abstract

Si‐based anodes (e.g., SiO_*x*_) can increase the specific energy of Li‐ion batteries but decrease cycle life due to volume change‐reasoned material stress, which requires continuous reformation of the solid electrolyte interphase (SEI) at the cost of active Li loss (ALL). Even in small ratios within a binary anode with graphite, Si can be exposed to a high state‐of‐charge as it can be stressed by transfer lithiation from lithiated graphite. SEI engineering via rational design of electrolyte additives can be an eligible measure, but is particularly challenging, as the SEI must effectively passivate and prevent detrimental SiO_*x*_|LiPF_6_ contact in order to suppress failure cascades caused by generated HF (e.g., electrode crosstalk), while efficiently minimizing ALL. Also, theoretically, the recovery of the “SEI waste” stemming from the continuous SEI reformation can be advantageous and, for example, be realized via a designed and controlled electrolyte solubility, where the respective diffusion toward the cathode would compensate ALL by oxidation reactions. Here, an easily modifiable B‐based compound (dioxazaborole) is synthesized and exemplarily investigated. While it forms a partly effective SEI and can be suitable for “SEI‐waste” recovery, the remaining challenges of gassing and resistance require further design optimization, consequently highlighting the difficulty of an “allrounder” additive.

## Graphical abstract

An ideal electrolyte additive, particularly for Si‐based anodes, should (i) promote the formation of an effective SEI, i.e., passivating and suppressing LiPF_6_/SiO_*x*_‐contact, (ii) minimize active Li loss (ALL) through efficient SEI formation, (iii) ideally enable “SEI‐waste” recovery and/or (iv) undergo sacrificial oxidation for prelithiation purposes, while minimizing resistance, gassing, self‐discharge, and costs.

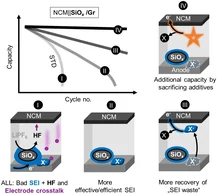

## 1 Introduction

The abundance of silicon, combined with its low (de)lithiation potential and ~10‐times higher theoretical specific capacity compared to state‐of‐the‐art (SOTA) graphite, renders it a promising anode material to increase the specific energy of lithium‐ion batteries (LIBs) [1, 2]. However, silicon suffers from large volume changes during charge/discharge cycling, which leads to, among others, particle cracking, pulverization, electronic contact loss, and severe capacity fade [3, 4, 5]. The latter can be predominantly attributed to solid electrolyte interphase (SEI) rupture and reformation, which consumes active Li (Li^+^ + e^−^) [6, 7], thus available capacity [8, 9, 10].

As a common countermeasure, pure Si‐ or SiO_*x*_ particles are mixed in small amounts (5–10 wt%) with graphite to form a binary active material composite (Figure 1a ) [13, 14, 15]. Though cycle life can be enhanced compared to pure Si anodes under common laboratory test protocols, the aforementioned challenges can still be problematic under real‐life application conditions, as the local material configuration within the Si/graphite or SiO_*x*_/graphite composite can overcharge Si during long durations in a fully charged state – a process known as “transfer lithiation” (Figure 1b) [11]. Here, more facile lithiation kinetics in graphite during charging can lead to a local element phenomenon, where the more lithiated graphite can continuously transfer charge/active Li (= Li^+^ + e^−^) to SiO_*x*_ due to differences in potential levels, which increases material stress and mechanical degradation [11].

**FIGURE 1:**
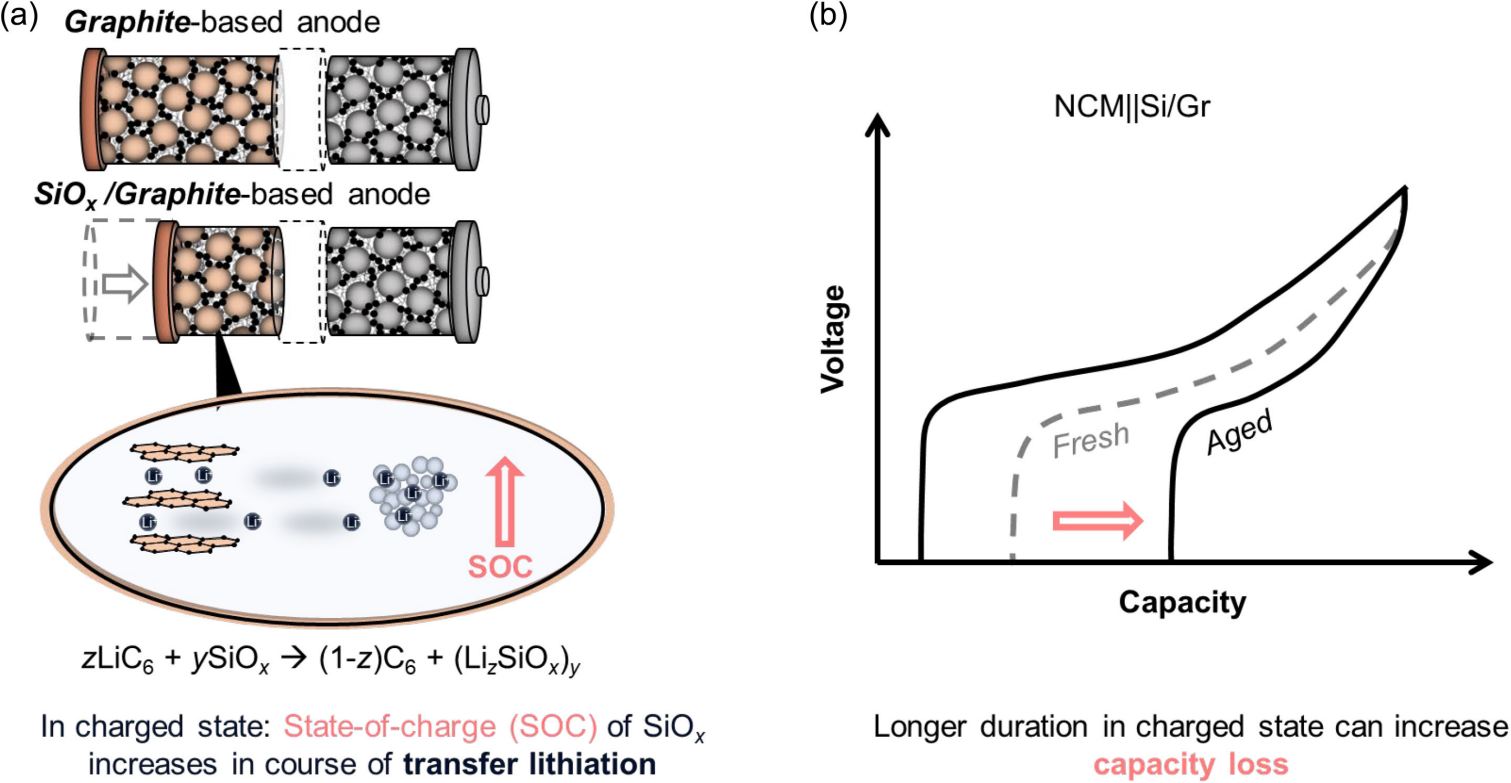
Schematic overview of LIBs with Si/Graphite‐based composite anodes, (a) which can minimize the cell volume and thus enhance the specific energy and energy density. However, Si can still obtain a high SOC over the course of transfer lithiation process, thereby suffering from material stress and capacity loss. (b) Consequently, a larger capacity loss after long‐duration storage can be expected, as illustrated by the cyclic voltage profile. Adapted from ref. [11] (panel (a), Copyright 2024, Wiley‐VCH GmbH) and ref. [12] (panel (b), Copyright 2026, Wiley‐VCH GmbH) under the Creative Commons Attribution License (CC BY 4.0).

An electrolyte that forms an effective SEI to accommodate the large volume changes of Si‐based particles remains a predominant R&D goal [10, 16]. The SEI formed by SOTA electrolyte formulations, i.e., LiPF_6_ in an ethylene carbonate (EC)–ethyl methyl carbonate (EMC) mixture (= STD), is known to be unstable and ineffective during charge/discharge cycling [4, 17]. This leads not only to active lithium loss (ALL) per se but also to HF generation through reactions of LiPF_6_ with SiO_*x*_ surface water or hydroxides [12]. The resulting HF can promote dissolution of transition metals (TMs) that damage the anode and the SEI in the course of electrode crosstalk (Figure 2, (I))—particularly literature‐known for high‐voltage LIBs [18, 19]—necessitating further electrolyte R&D [10, 16, 20, 21, 22, 23].

**FIGURE 2:**
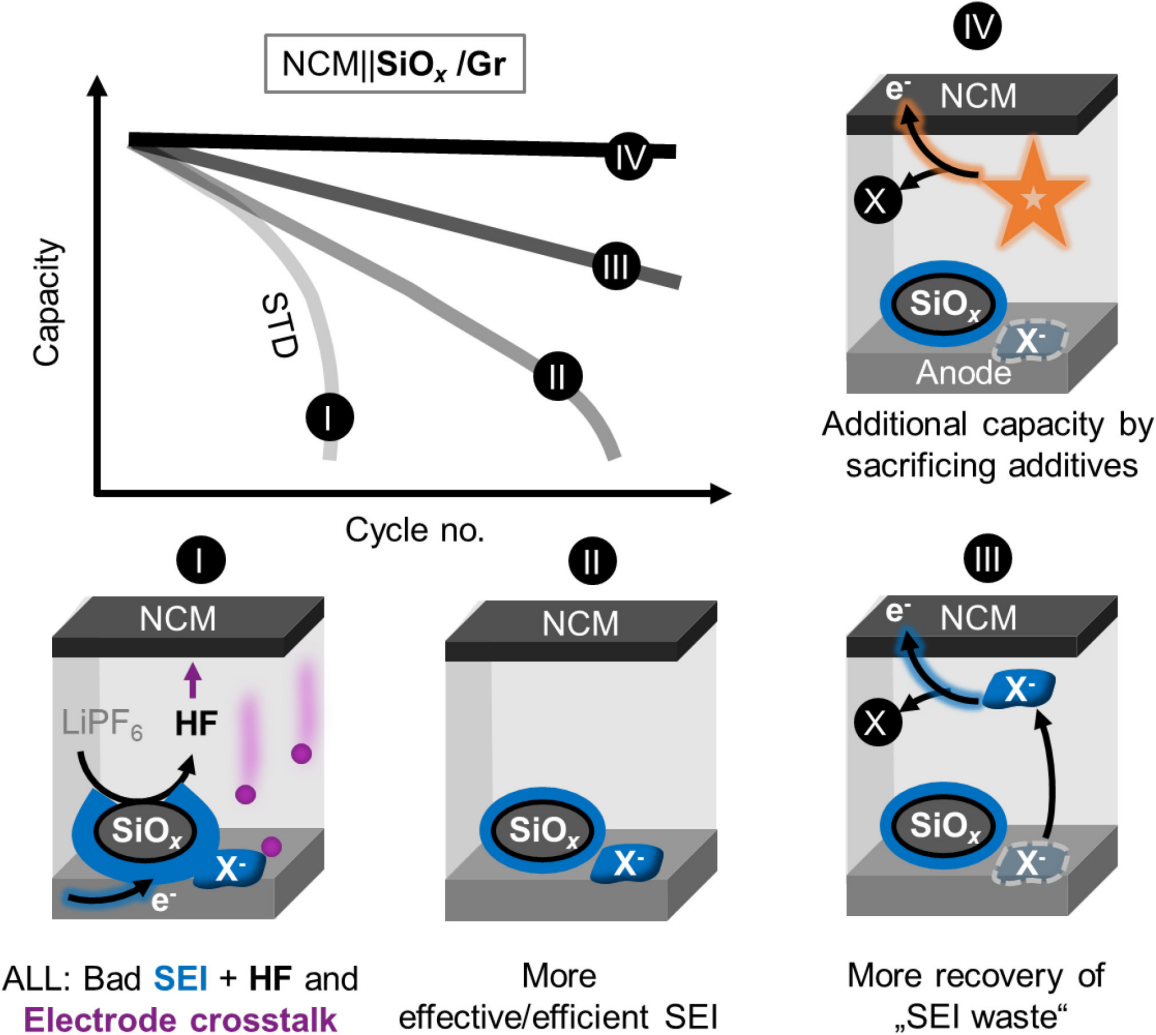
(I) With conventional Si‐based anodes, capacity loss and fading during cycle life are high due to active Li loss (ALL) in the course of SEI reformation. In STD electrolytes, the ineffective SEI can enable detrimental LiPF_6_ contact with SiO_*x*_ and trigger HF‐induced electrode crosstalk, which additionally increases ALL. (II) Increasing the effectiveness and efficiency of SEI formation by addition of, e.g., FEC can suppress these failure cascades, as long as the additive is not consumed. (III) The inevitable reformation of SEI in the course of Si‐based volume changes and subsequent enrichment in “SEI waste” can be theoretically counteracted by a tailored SEI design with adjusted solubility of SEI waste. This way, the formerly reduced SEI species can diffuse to the cathode and undergo oxidation, potentially compensating ALL, recovering cell capacity, and ultimately prolonging cycle life. (IV) To finally compensate remaining capacity losses, an oxidatively unstable electrolyte additive can be incorporated, purposely being oxidized and thus providing relithiation capacity, a strategy known in the literature as the use of a “sacrificing additive”. Adapted from ref. [12] (Copyright 2026, Wiley‐VCH GmbH) under the Creative Commons Attribution License (CC BY 4.0).

Optimizing electrolyte functions via additives is promising as it is practically eligible, economical, and scalable while being effective in improving electrochemical performance without sacrificing bulk properties of the SOTA electrolyte [24, 25]. Typically, cyclic carbonates such as vinylene carbonate (VC) or fluoroethylene carbonate (FEC) are mixed in small amounts (1–5 wt%) as they are reduced before other electrolyte components, e.g., EC [26, 27, 28, 29]. The FEC is favored and can be regarded as a benchmark additive due to the generally better cycle life, higher oxidative stability, a more effective and low‐resistive SEI, low and mostly reversible self discharge, negligible gassing, and relatively low ALL [30, 31]. Nevertheless, FEC gets continuously consumed over the course of charge/discharge cycling and can thus delay, but not eliminate, end‐of‐life (EOL), i.e*.*, the onset of characteristic rollover fading driven by the aforementioned failure cascades (Figure 2, (II)). Increasing the amount of FEC, e.g., as a cosolvent, would further increase costs.

Alternatively, the SEI can theoretically counteract ALL by capacity/active Li (AL) recovery via diffusion of partly dissolved inactive SEI species toward the cathode and subsequent oxidation reactions, as assumed in electrolytes with oxalatoborate‐based Li salts, e.g., lithium bis(oxalato)borate (LiBOB, Figure 2, (III)) [12]. However, these salts are oxidatively less stable, less conductive than LiPF_6,_ and additionally tend to generate gas during decomposition. When keeping the SOTA basic electrolyte, the ideal electrolyte additive needs to (1) be effective (e.g., passivation, no SiO_*x*_|LiPF_6_ contact), (2) be efficient (minimize ALL), (3) minimize resistance (rises), (4) minimize (irreversible) self‐discharge (SD), (5) recover capacity via inverse crosstalk of inactive SEI (“self‐healing”), (6) minimize gas evolution, and (7) be cost‐efficient.

For systematic R&D toward understanding structure–property relations, easily‐modifiable additive structures are necessary. Molecules such as *O*‐carboxyanhydrides, *N*‐carboxyanhydrides, and dioxazolones are known to be beneficial for effective SEI formation and also offer good modification possibilities. However, their application‐oriented synthesis is known to be expensive and demanding in terms of handling and safety [16, 20, 32, 33]. Besides that, boron‐based compounds are concluded to increase the capacity recovery via inverse crosstalk [12]. Consequently, novel additive classes that integrate established organic SEI‐forming backbones with boron‐containing functional groups may provide access to a dynamic SEI design concept, in which robust anode passivation is combined with active‐lithium recovery through inverse crosstalk and, where applicable, sacrificial additive oxidation.

In this study, 2,5‐diphenyl‐1,3,4,2‐dioxazaborole (DOB, Figure 3) is investigated as an electrolyte additive in NCM 523 || 10% SiO_*x*_–graphite cells for the first time not only as an SEI additive to increase efficiency but also as the basis of boron‐based additive structures toward R&D of “self‐healing” SEI. Moreover, according to the ab initio calculated energetic level of the highest occupied molecular orbital (HOMO, see Section 2), it can also strategically act as a “sacrificing additive” for prelithiation purposes in order to additionally compensate ALL (Figure 2, (IV)) [34].

**FIGURE 3:**
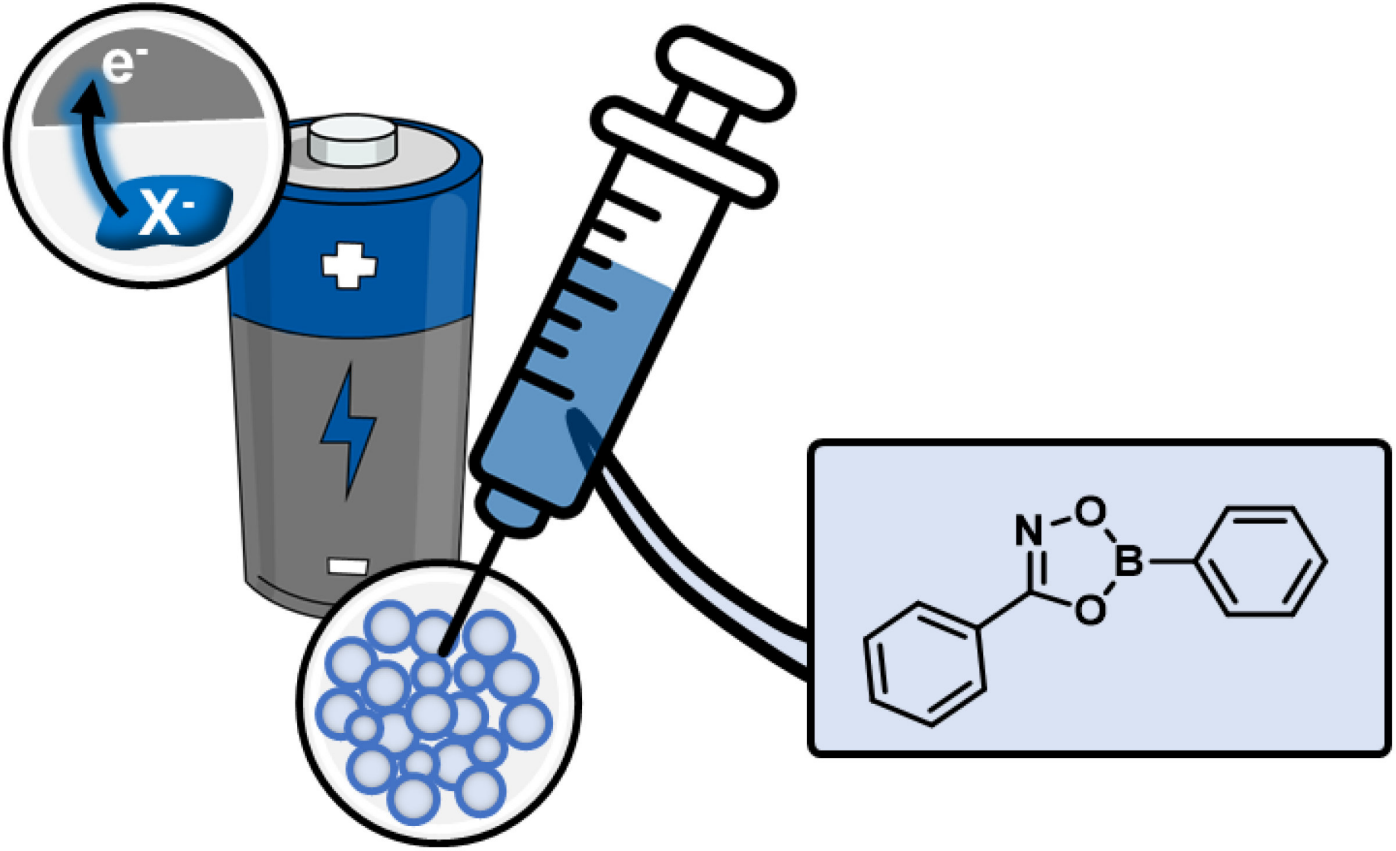
Chemical structure of DOB. Illustrations adapted from ref. [11] (Copyright 2024, Wiley‐VCH GmbH) under the Creative Commons Attribution License (CC BY 4.0).

Building on prior insights into the beneficial SEI‐forming behavior of dioxazolones, the design rationale for DOB derives from the established reductive pathways of dioxazolone additives. Their electrochemical decomposition is known to generate lithium carbonate via CO_2_ formation in combination with organic polymeric species, the latter contributing to both mechanically and chemically resilient SEI architectures [33, 35]. Considering that carbonyl functionality in dioxazolones can be systematically replaced by monosubstituted borane units—and that boron‐based species have been associated with enhanced SEI stability [36, 37] and improved capacity recovery via inverse crosstalk—the synthesis of DOB is guided by the hypothesis that its electrochemical fragmentation would yield organic polymeric frameworks plus boron‐containing compounds or salts as complementary contributors to an effective SEI and long cycle life. Given that the primary aim of this proof‐of‐concept study is to evaluate the dioxazaborole motif as a new boron‐containing electrolyte‐additive scaffold, phenyl groups are selected as synthetically straightforward reference substituents, allowing access to the target compound from *N*‐hydroxybenzamide and phenylboronic acid without introducing additional functionalities specifically designed to undergo reductive decomposition.

This additive can be efficiently synthesized in a single step from *N*‐hydroxybenzamide (readily accessible from benzoyl chloride) and phenylboronic acid, generating DOB in a high yield (65%). The removal of water from the reaction system via the well‐established toluene/water azeotrope system, followed by purification via recrystallization from a minimal amount of ethanol, highlights the potential suitability of this method for large‐scale industrial applications. Based on the purchase cost of the reactants and the achieved yield [38, 39], the feedstock cost of DOB is estimated to be only USD 2.8 per  kg.

## 2 Results and Discussion

### 2.1 SEI Formation and Electrochemical Performance

Figure 4a presents the 0th cycle (“formation cycle”) differential capacity versus voltage plots of NCM 523 || 10% SiO_*x*_–graphite cells with electrolyte blends listed in Table 1, providing insight into the initial reduction processes governing SEI formation. Cells with the STD electrolyte (1 M LiPF_6_ in an ethylene carbonate (EC)–ethyl methyl carbonate (EMC) solvent mixture) exhibit a sharp peak at 0.7 V versus Li|Li^+^, which originates from EC reduction and represents the baseline SEI formation pathway [40, 41]. In contrast, a broader peak at 0.8 V versus Li|Li^+^ arising from fluoroethylene carbonate (FEC) reduction is observed in cells with the STD + FEC electrolyte, notably suppressing EC reduction [10, 16]. This behavior is consistent with the well‐established role of FEC as a preferentially reduced additive that enables controlled and efficient SEI formation and thus serves as a benchmark for conventional SEI‐forming strategies. Adding 2,5‐diphenyl‐1,3,4,2‐dioxazaborole (DOB) to the STD electrolyte results in multiple reduction peaks roughly at 1.4, 1.1, and 0.9 V versus Li|Li^+^. Compared to FEC, the significantly earlier onset and multi‐step reduction behavior indicate a fundamentally different reduction pathway, suggesting that DOB participates more extensively and reactively in the initial interphase formation. DOB appears to promote a more complex and compositionally diverse SEI. The multi‐step SEI formation process shows that the SEI formed by the first two reduction products is not passivating, necessitating a third reduction step to form a passivating layer. Similar peaks at 1.4 and 1.1 V versus Li|Li^+^ are also observed in cells with the STD + DOB‐FEC electrolyte, though there is an additional peak at 0.75 V versus Li|Li^+^, which can originate from either DOB or FEC reduction. The three reduction peaks here further support the previous argument that the first two reduction products are poorly passivating.

**FIGURE 4:**
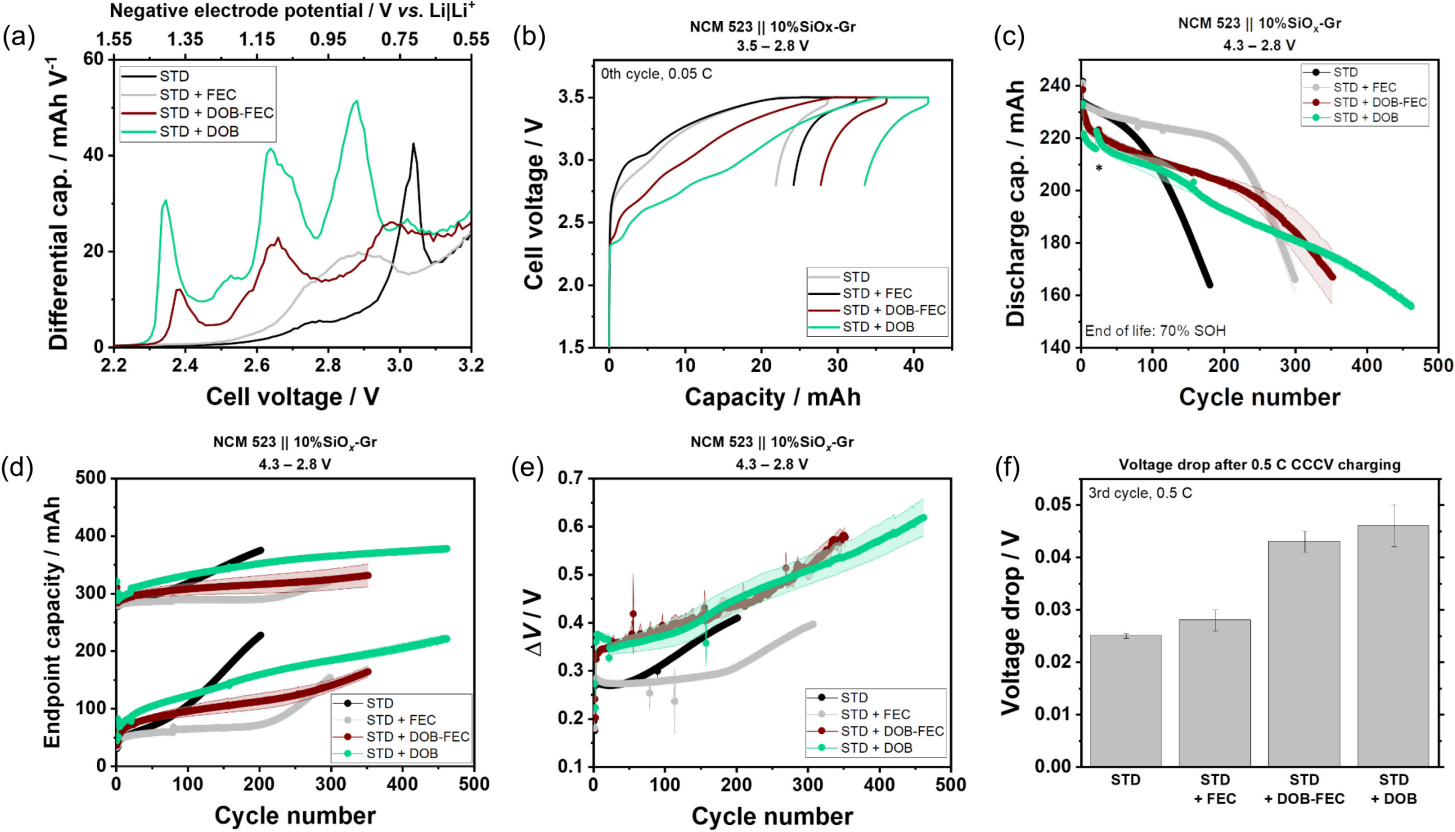
(a) Differential capacity versus voltage plot, (b) 0th cycle voltage versus capacity, (c) discharge capacity versus cycle number plot, (d) capacity endpoint slippage, (e) cell polarization versus cycle number plot, and (f) voltage drop after 0.5 C CCCV charging step in the 3rd cycle of cells with the STD, STD + FEC, STD + DOB‐FEC, and STD + DOB electrolytes. The asterisk sign (*) in (c) indicates battery cycler shutdown, causing the capacity anomaly. STD and STD  + FEC data were adapted from ref. [12] (Copyright 2026, Wiley‐VCH GmbH) under the Creative Commons Attribution License (CC BY 4.0).

**TABLE 1:** Detailed composition and nomenclature of electrolytes in this study. The additive amount of 0.19 mmol g^−1^ electrolyte is based on 2 wt% of FEC [10, 32].

Electrolyte	Additive	Molecular wt.,g mol^−1^	Amount, mmol g^−1^_electrolyte_	Amount, wt%	Name
1 M LiPF_6_ in EC:EMC (3:7 by weight)	—	—	—	—	STD
	FEC	106.05	0.19	2	STD + FEC
	DOB‐FEC	1:1 molal ratio	0.19	2.1 and 1	STD + DOB‐FEC
	DOB	223	0.19	4.2	STD + DOB

As presented in Figure 4b, cells with STD + FEC exhibit lower formation cycle capacity losses (0th cycle; see Figure S1) compared to STD, reflecting the well‐established efficiency of FEC in forming a stable and low‐resistive SEI, while STD + DOB‐FEC and STD + DOB show increased capacity losses during SEI formation (Figures 4b,c and S2a) [10, 16]. Cells with STD + DOB have ≈10 mAh lower initial capacity compared to the STD or STD + FEC (Figures 4c and S2b); nevertheless, cells with DOB have a slightly longer cycle life.

The discharge endpoint capacity versus cycle number plot (Figure 4d) is highest for STD + DOB, indicating more ALL due to the reduction reaction. Nevertheless, the simultaneously elevated charge endpoint slippage indicates oxidative reactions and ALL compensation. This oxidation reaction may originate from soluble SEI speciesthat can diffuse toward the cathode and recover lost electrons (and generate AL) via oxidation, a process described in the literature as SEI “self‐healing” [19, 42]. Alternatively, the DOB itself may be directly oxidized given its low oxidative stability limit (Figure 5d). Or it triggers more NCM delithiation due to high ALL in the course of upper potential shifts. In the initial 40 cycles (cycle 4–43) of cells with STD + DOB and STD + DOB‐FEC, the oxidation reactions contribute to ≈0.63 mAh/cycle and ≈0.39 mAh/cycle, respectively. Notably, these trace amounts of capacity can be meaningful for improving capacity retention but are not enough to compensate for ALL during SEI formation (≈10 mAh more for STD + DOB vs. STD), under standard formation conditions. When normalized to the amount of DOB in the electrolyte, DOB contributes to 17.8 ± 0.6 $\mathrm{mAh}{\text{ cycle}}^{\text{−1}}\;g_{\text{DOB}}^{-1}$ and 19.6 ± 4.9 $\mathrm{mA}h{\text{ cycle}}^{-1}{\text{ g}}_{\text{DOB}}^{\text{−1}}$ in cells with STD + DOB and STD + DOB‐FEC, respectively. In the later cycles (cycle 313–352) of cells with STD + DOB and STD + DOB‐FEC, the oxidation reactions contribute to ≈0.07 mAh/cycle and ≈0.14 mAh/cycle, respectively. The initially similar value of normalized charge slippage and the faster decay of charge slippage in cells with STD + DOB hint that the oxidation reaction originates mostly from direct DOB oxidation. Considering the larger amount of DOB consumption for SEI formation, DOB is depleted faster, thus contributing less to charge slippage in the later cycles in cells with STD + DOB versus STD + DOB‐FEC.

**FIGURE 5:**
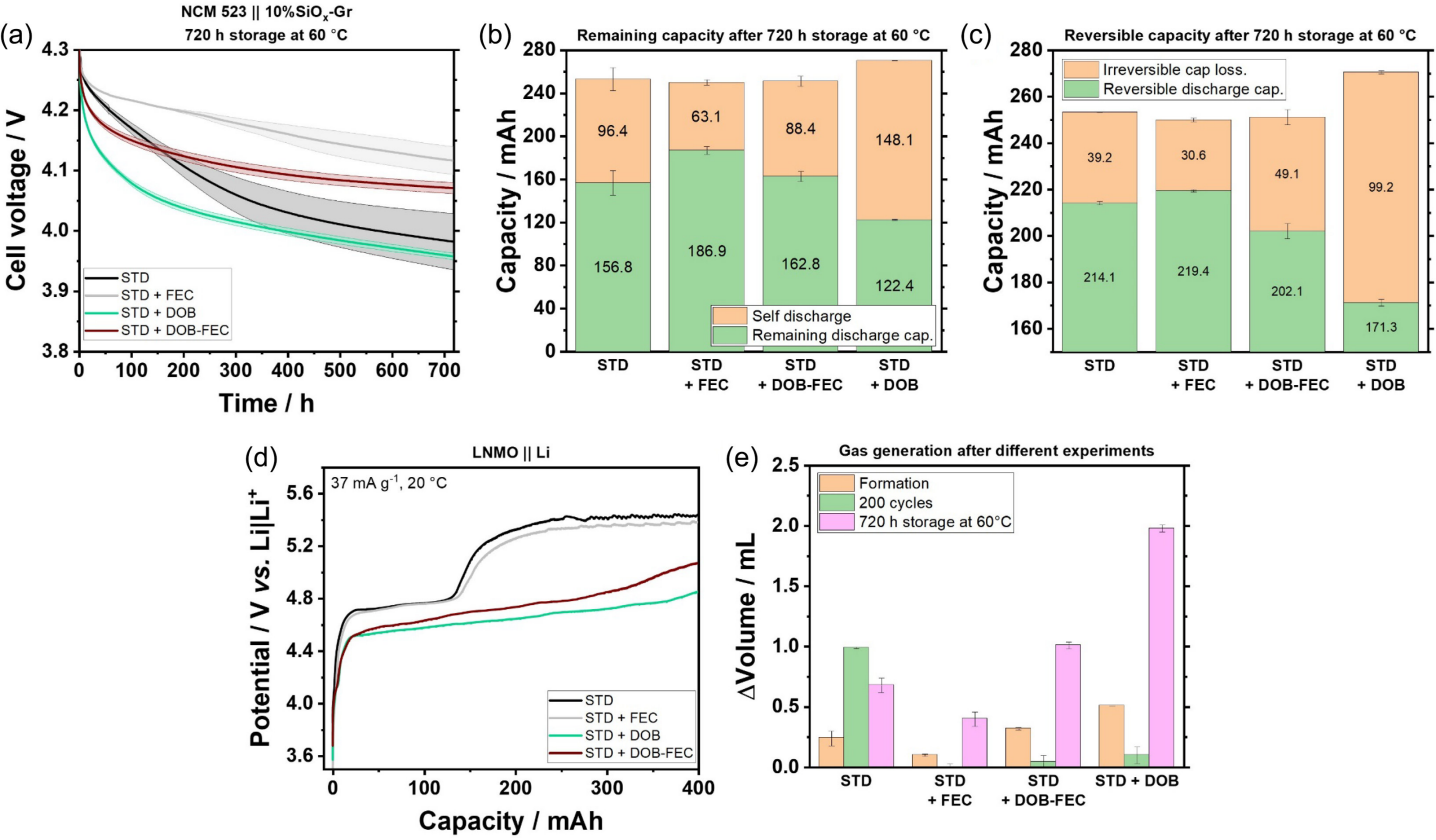
(a) Cell voltage versus time plots, (b) self‐discharge and remaining discharge capacities, (c) irreversible capacity losses and reversible discharge capacities, (d) galvanostatic overcharge experiments, (e) gas generation after formation cycles, 200 charge/discharge cycles, and 720 h storage at 60 °C of cells with the STD, STD + FEC, STD + DOB‐FEC, and STD + DOB electrolytes. STD and STD +   FEC data were adapted from ref. [12] (Copyright 2026, Wiley‐VCH GmbH) under the Creative Commons Attribution License (CC BY 4.0).

In the case of cells with STD + FEC, the discharge endpoint slippage is initially low (≈0.26 mAh/cycle) but then increases abruptly after 200 cycles, indicating FEC depletion [12]. In contrast, the slippage of cells with STD + DOB is consistently high (≈0.85 mAh/cycle). The absence of an abrupt change in slippage of these cells indicates that the DOB has not been fully consumed by the time the cells reach 70% SoH (EOL). Considering the multi‐step reduction process of DOB (Figure 4a), it is possible that less DOB is consumed than FEC, despite the larger discharge slippage observed for DOB. These differences in the additive consumption, combined with the AL generation from oxidation, result in the differing capacity fade behavior of cells with and without DOB.

At the same time, as shown in Figure 4e, DOB‐based cells exhibit higher initial voltage hysteresis (Figure S2c) and increased polarization growth during charge/discharge cycling, as well as a larger voltage drop after the constant current‐constant voltage (CCCV) charging step (Figure 4f). These features reflect a more resistive interphase with altered transport properties compared to the FEC‐derived SEI [43]. These characteristics are not unexpected but represent an inherent consequence of the increased reactivity and compositional complexity of the DOB‐derived interphase.

### 2.2 SD and Gassing Behavior

Figure 5a displays the open‐circuit voltage (OCV) decay of fully charged cells (4.3 V), reflecting the SD behavior over 720 h of storage at 60 °C. The OCV of cells containing FEC remains constant compared to STD, indicating low SD. In contrast, DOB‐containing electrolytes exhibit more pronounced SD and higher irreversible capacity losses after storage (Figure 5b,c) [10]. Although the large SD may be caused by the lower anodic stability of DOB (Figure 5d) that can cause oxidation reactions, the large irreversible capacity loss of cells with STD + DOB (Figure 5c) hints that reduction reactions play a more dominant role in causing the SD.

The poorer stability of DOB‐derived SEI is further reflected in increased gas generation after 720 h of storage at 60 °C (Figure 5e), especially for cells with STD + DOB. In the case of STD + DOB‐FEC, SEI stability can be improved; thus, irreversible capacity loss and gassing are also lowered. While elevated gas formation is also observed after formation cycles, consistent with the higher charge consumption during initial SEI formation, only minor gas evolution occurs during 200 charge/discharge cycles. This indicates that under regular cycling conditions, the DOB‐derived SEI is relatively stable, and the oxidation reaction occurring at every charge cycle produces only low amounts of gas.

### 2.3 Density Functional Theory (DFT) Calculations

The low stability of DOB compared to that of other electrolyte components is shown in Figure 6. The ab initio calculated energy level of the HOMO of DOB is higher compared to the SOTA solvents and additives such as EC, EMC, VC, and FEC, as well as conventional salts like LiPF_6_, LiDFOB, and LiBOB. This is in line with the experimentally observed low oxidation potential of DOB (Figure 5d), rendering DOB as a potential sacrificial additive, as discussed above. Also, the lowest unoccupied molecular orbital (LUMO) is in line with the experimentally observed high reduction potential (Figure 4a), i.e*.*, reduction before the other components, thus predominantly impacting the SEI chemistry.

**FIGURE 6:**
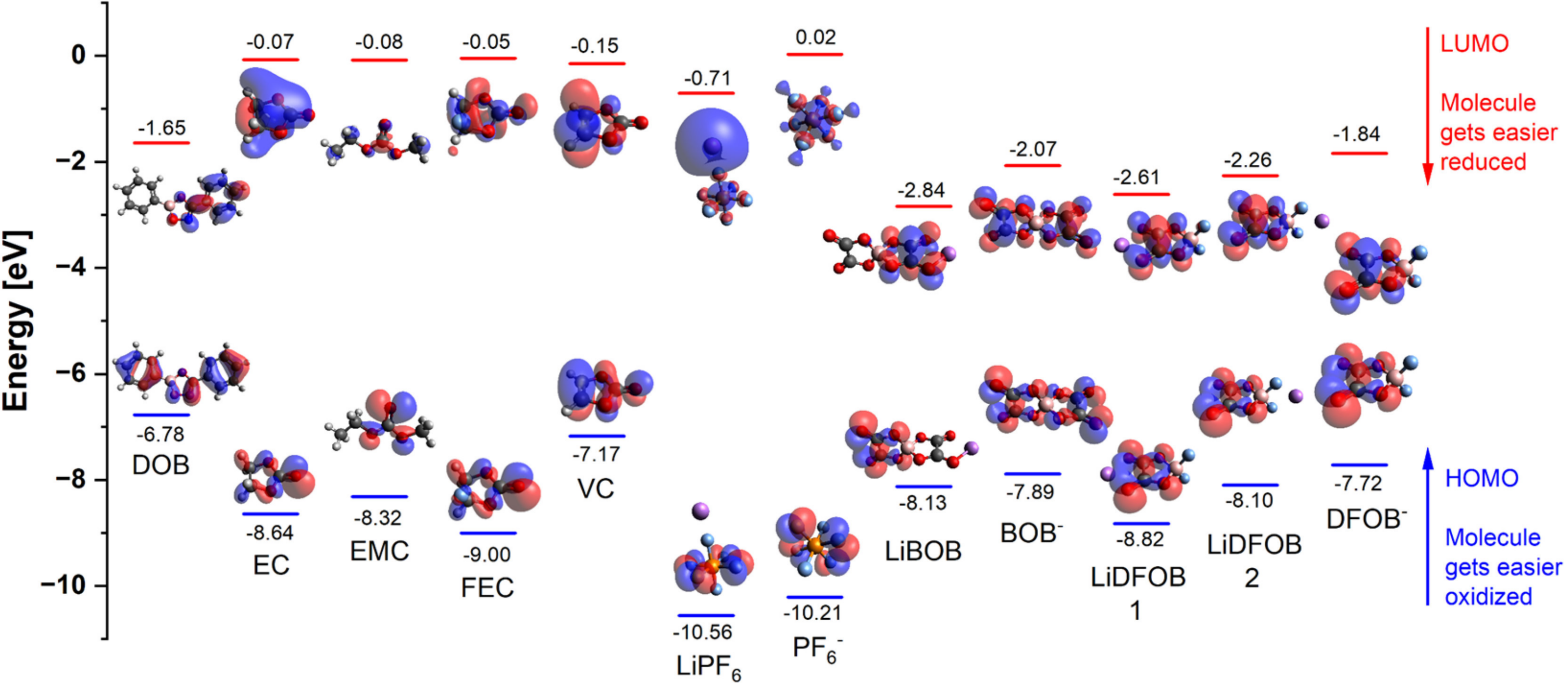
Calculated HOMO and LUMO energy values for DOB and various other electrolyte solvents, additives and salts. Reference compound data were adapted from ref. [12] (Copyright 2026, Wiley‐VCH GmbH) under the Creative Commons Attribution License (CC BY 4.0).

### 2.4 SEI Investigation

Scanning electron microscopy (SEM) images of SiO_*x*_–graphite negative electrodes after 200 charge/discharge cycles are presented in Figure 7. SiO_*x*_ particles are identified (yellow circles) via energy‐dispersive X‐ray spectroscopy (EDX). In cells with the STD electrolyte, these particles are visually covered (Figure 7a), likely by SEI, TM deposits (Figure 7e), and high surface area lithium (HSAL), as discussed in previous reports [10, 16].

**FIGURE 7:**
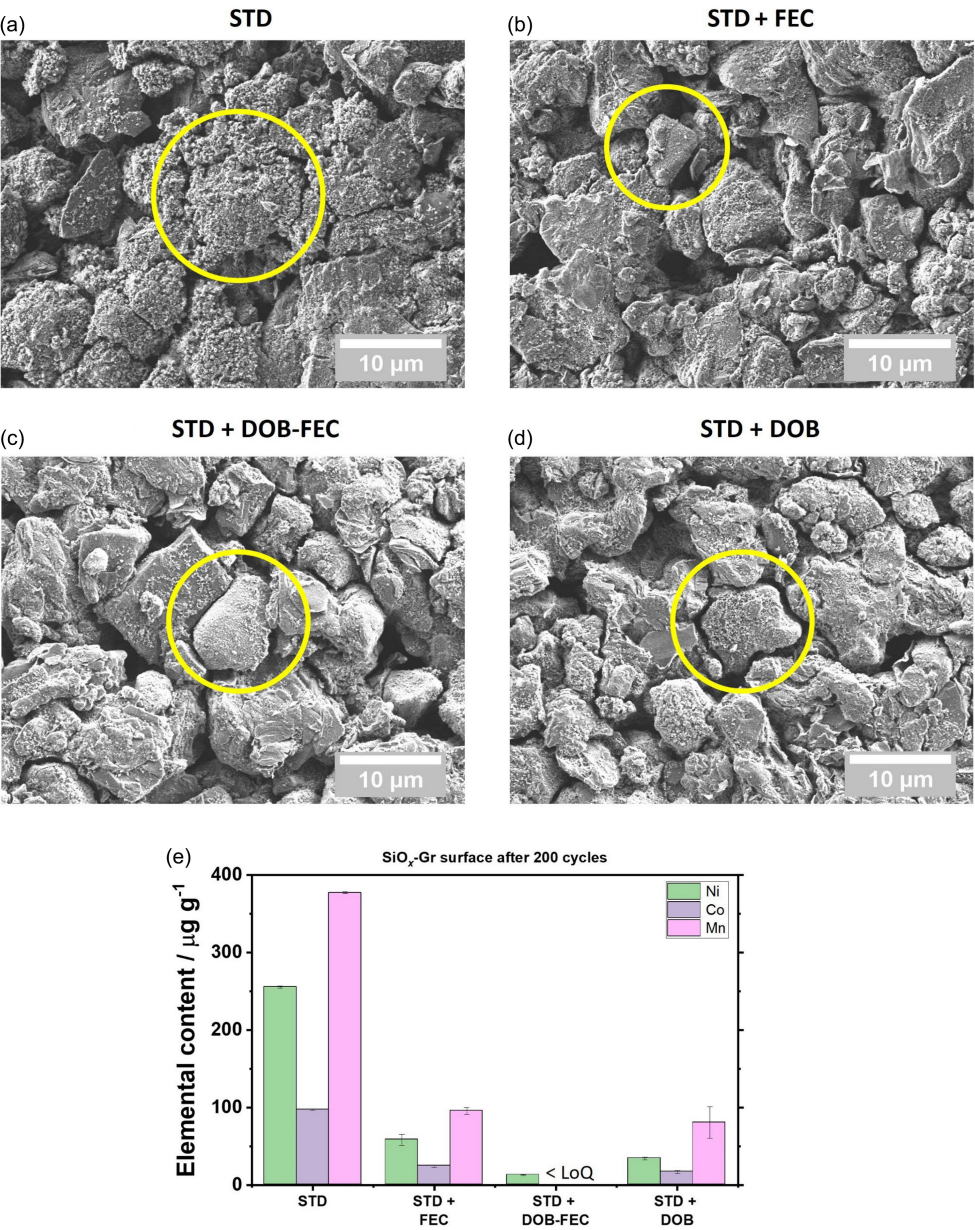
SEM images of negative electrodes extracted from cells with the (a) STD, (b) STD + FEC, (c) STD + DOB‐FEC, and (d) STD + DOB electrolytes after 200 charge/discharge cycles. The yellow circles indicate the SiO_*x*_ particles, identified via EDX. (e) TM elemental content on SiO_*x*_–Gr negative electrodes after 200 cycles, obtained via ICP‐OES. The Co and Mn amounts are below the limit of quantification (LoQ) in the case of STD + DOB‐FEC electrolyte. (a), (b), and parts of (e) were adapted from ref. [12] (Copyright 2026, Wiley‐VCH GmbH) under the Creative Commons Attribution License (CC BY 4.0).

In contrast, electrodes cycled with additive‐containing electrolytes (Figure 7b–d) show a visibly reduced coverage of SiO_*x*_ particles, indicating a modified interfacial environment. In particular, the decreased presence of TM deposits in these systems can be attributed to the suppression of detrimental SiO_*x*_–LiPF_6_ interactions. Such interactions are known to initiate autocatalytic LiPF_6_ decomposition by water generation (Equation I), leading to more HF formation and subsequent TM dissolution from the cathode, which contributes to electrode crosstalk [44, 45, 46]. Consequently, the reduced TM deposition confirms an overall improvement in SEI effectiveness compared to the STD electrolyte in this aspect [31, 47].

(I) $${\mathrm{SiO}}_{2}+4\mathrm{HF}\to {\mathrm{SiF}}_{4}+2H_{2}O$$

Time‐of‐flight secondary ion mass spectrometry (ToF‐SIMS) depth profiling of anodes after 200 cycles provides details on the chemical composition and distribution of the SEI (Figure 8). In the STD electrolyte, the gradual decay of Li^−^ and F_2_Li^−^ intensities with sputter depth indicates an interphase rich in LiPF_6_ decomposition products (Figure 8a). In contrast, STD + FEC shows a more rapid decay of these fragments (Figure 8b), consistent with a more compact and efficient SEI, which is less LiPF_6_‐derived, in line with the lower charge consumption during SEI formation as well as previous reports [12]. Despite a comparable presence of organic nonvolatile species (e.g., C_2_H^−^ fragments) in STD and STD + FEC electrolytes, the FEC‐derived SEI is generally considered to be more crosslinked and elastomeric, enabling improved mechanical stability during SiO_*x*_ volume changes [48, 49, 50]. In contrast, the SEI formed in DOB‐containing electrolytes exhibits distinct chemical characteristics (Figure 8c,d). For STD + DOB and STD + DOB‐FEC, characteristic fragments such as BO^−^, CNC_6_H_5_O^−^, and LiCO_3_^−^ are observed with higher relative intensities, evidencing the incorporation of DOB‐derived species into the interphase. Notably, these fragments persist over larger sputter depths in STD + DOB‐FEC, indicating that DOB‐derived species are not only formed but also retained within the interphase [48]. Furthermore, the comparatively slow decay of F_2_Li^−^ fragments in STD + DOB‐FEC suggests the formation of a thicker SEI. For STD + DOB, a faster decay of Li^−^ and F_2_Li^−^ fragments is observed compared to STD despite the higher charge consumption during SEI formation (Figure 4a,b).

**FIGURE 8:**
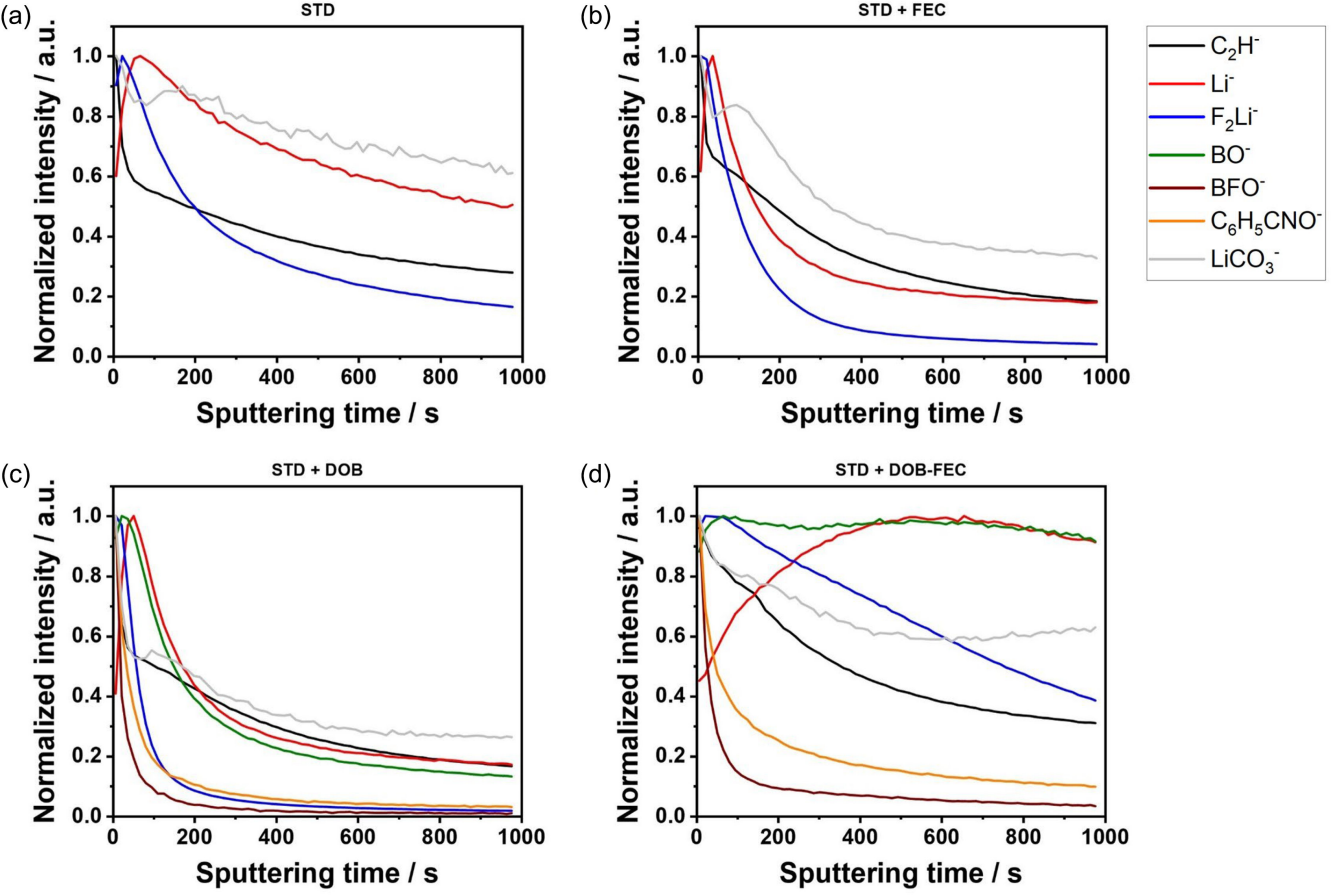
Sputter depth profiles of washed negative electrode surface extracted from cells with the (a) STD, (b) STD+FEC, (c) STD+DOB, and (d) STD+DOB‐FEC electrolytes, obtained via ToF‐SIMS.

The thinner‐than‐expected SEI thickness for STD + DOB points to SEI dissolution, but the dissolved products are unlikely to contribute to AL recovery via an oxidation reaction, as discussed previously in the electrochemical performance section. The thicker SEI for STD + DOB‐FEC suggests less pronounced SEI dissolution due to the “molecular cling effect” of FEC [51], and consequently lower charge endpoint slippage (Figure 4d) and less irreversible capacity loss and gassing under high‐temperature storage (Figure 5c,e) compared to the STD + DOB electrolyte. The higher charge endpoint slippage of STD + DOB‐FEC versus STD + FEC indicates that oxidation reactions still occur despite the limited SEI dissolution, further suggesting that DOB contributes to the oxidation reactions.

Complementary laser‐induced breakdown spectroscopy (LIBS) measurements of the ⌀12 mm SiO_*x*_ *–*graphite negative electrodes (punched out from the pouch cell jelly roll) further confirm the presence and spatial distribution of boron across the anode surface in DOB‐containing systems (Figure 9). The spatial distribution of boron across the electrode indicates that DOB‐derived species are not confined to localized regions but contribute to a broadly distributed interphase. This observation supports the notion that DOB is involved in SEI formation rather than acting as a minor or secondary component.

**FIGURE 9:**
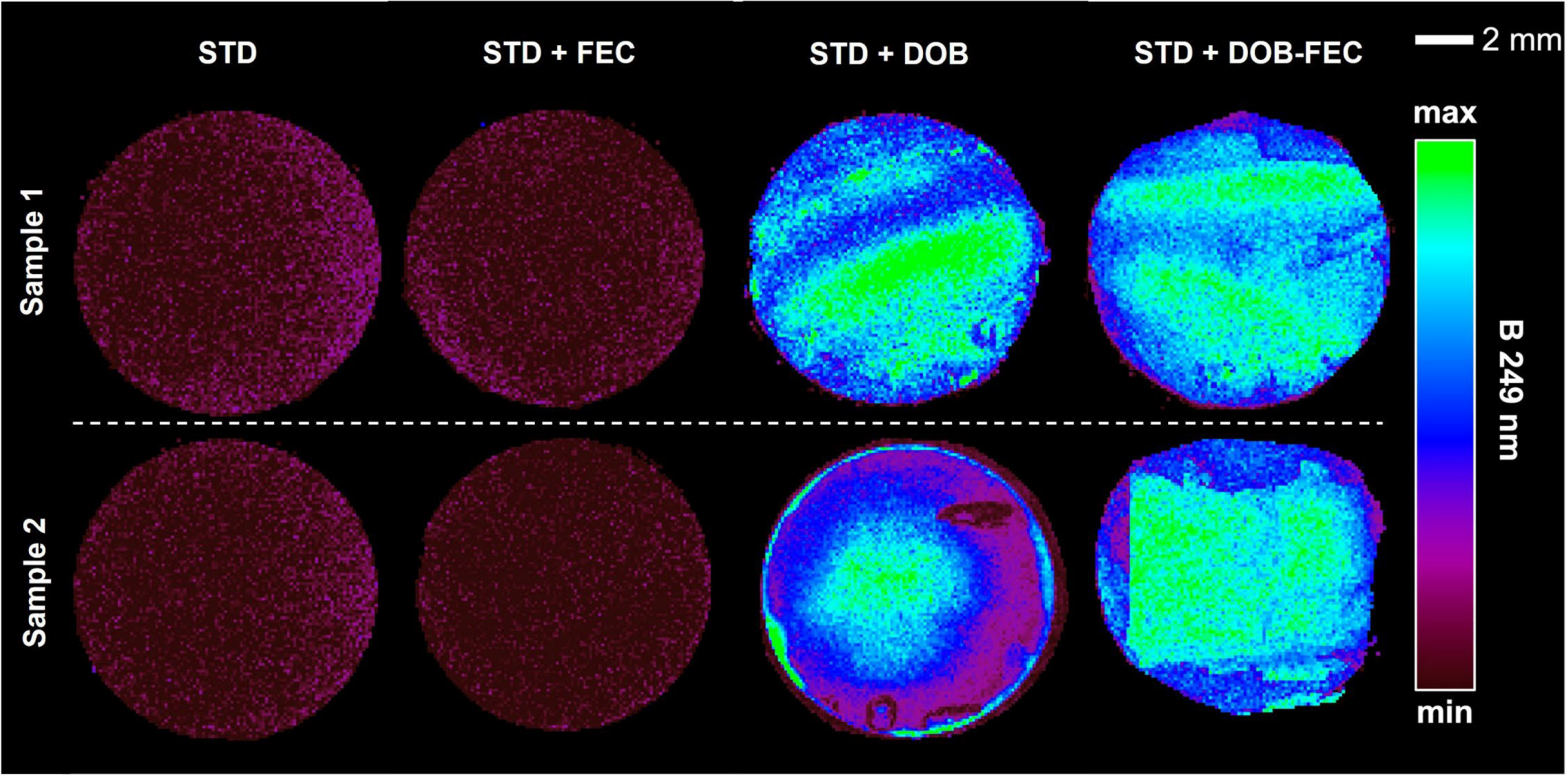
Duplicate LIBS maps of negative electrodes extracted from cells with the STD, STD + FEC, STD + DOB, and STD + DOB‐FEC electrolytes after formation cycles. Despite the differences in elemental distributions, likely due to pressure inhomogeneity in different parts of the jelly roll [19, 52], boron is clearly detected in all DOB‐based samples. The abrupt change in intensity for the bottom STD + DOB‐FEC sample stems from electrode coating delamination.

Overall, the combined ToF‐SIMS and LIBS results demonstrate that DOB does not merely modify the extent of SEI formation but fundamentally alters its chemical composition and spatial distribution. In contrast to the compact and predominantly passivating SEI formed by FEC, the DOB‐derived interphase is characterized by the incorporation of boron‐containing species, increased compositional complexity, and indications of structural heterogeneity. The characteristic ToF‐SIMS fragments BO^−^ and CNC_6_H_5_O^−^ are indicators that DOB undergoes reductive ring‐opening/fragmentation during SEI formation, generating boron–oxygen species and phenyl‐containing organic fragments. Considering the multi‐step SEI formation in the case of the STD + DOB electrolyte (Figure 4b), the strong presence of BO^−^ in the SEI, and the passivating property of CNC_6_H_5_O^−^ based on dioxazolone additives [53], it is likely that these two products are formed in the last reduction step during SEI formation, forming a passivating SEI layer. That said, to improve the performance, additives should be tailored to produce these fragments. Adjusting the formation protocol is unlikely to be beneficial, as the first two nonpassivating products will always be formed.

## 3 Conclusions

SiO_*x*_ incorporation into graphite‐based anodes can increase the specific energy and energy density of Li‐ion batteries (LIBs), but at the cost of cycle life due to SiO_*x*_ volume change‐reasoned material stress over the course of charge/discharge cycling. This requires continuous reformation of the SEI, which consumes active lithium (AL), thus reducing capacity. The focus of electrolyte additive R&D is consequently set on an effective SEI, which is passivating and suppresses LiPF_6_ contact. The latter would otherwise react and produce HF, which can cause failure cascades by, e.g*.*, triggering metal dissolution from the cathode and initiating crosstalk. Further, the SEI needs to be efficient and decrease AL loss (ALL) but also decrease resistance, gas evolution, and SD. In theory, an SEI with “self‐healing” features would be advantageous as well, where a pronounced part of the futile (formerly reduced) SEI species (“SEI waste”) can diffuse toward the cathode and recover the capacity via an oxidation reaction, consequently prolonging the cycle life by compensating ALL. This is especially relevant for Si‐based anodes, which are regarded as producing relatively high amounts of SEI waste. This motivates a particular SEI design concept, in which electrolyte additives are not only optimized for finite anode passivation but are tailored to combine interphase stabilization with capacity recovery via inverse crosstalk and, optionally, additional additive oxidation for active‐lithium compensation (prelithiation via “sacrificing additives”).

Based on the established use of dioxazolones as electrolyte additives, the carbonyl group is replaced by a B–R unitto form dioxazaboroles, a novel class of electrolyte additives with promising synthetic accessibility, cost, and opportunities for systematic modification. The boron‐core is aimed to increase the “self‐healing” mechanism and is representatively introduced in this work by 2,5‐diphenyl‐1,3,4,2‐dioxazaborole (DOB), reported for the first time as an electrolyte additive.

When added to a SOTA electrolyte (1 M LiPF_6_ dissolved in an ethylene carbonate (EC)/ethyl methyl carbonate (EMC) solvent mixture), DOB is involved in the SEI formation, as shown by its early reductive decomposition at ~1.4 V versus Li|Li^+^, distribution of boron‐containing species over the anode surface LIBS, and presence throughout the SEI ToF‐SIMS. Although the presence of DOB slightly lowers the cell capacity (up to 10 mAh) compared to fluoroethylene carbonate (FEC) as the SOTA additive, DOB is able to suppress the abrupt “rollover failure” fading and prolong cycle life. With DOB, the absence of residuals on SiO_*x*_ particles SEM and the suppressed TM deposits on the anode inductively coupled plasma‐optical emission spectroscopy (ICP‐OES) indicate suppressed electrode crosstalk, suggesting DOB’s SEI effectiveness. In addition, a higher charge endpoint slippage indicates more oxidative reactions, thus a potential capacity‐recovering self‐healing mechanism, even without pronounced gassing during charge/discharge cycling. In contrast to the initial aim of producing SEI waste that can be oxidized in the cathode, it is discovered that DOB itself is likely the major species participating in the oxidation reaction or simply enhances cathode delithiation by upper potential shifts. Overall, DOB can be classified as a film‐forming additive, similar to FEC, and its low oxidative stability limit also warrants it being classified as a sacrificing additive.

Despite the benefits, the higher initial capacity loss (= lower 1st cycle Coulombic efficiency), resistance rise during charge/discharge cycling, (irreversible) SD, as well as gassing during formation and elevated temperature storage render DOB unsuitable for practical application. Furthermore, the dual‐additive approach combining DOB with FEC is not promising. Despite these limitations, the low oxidative stability limit of DOB may be utilized for pre‐lithiation purposes as it can be sacrificed during the initial charge process to generate an AL reservoir. More broadly, the present study demonstrates that boron‐containing additives can be designed to combine effective SEI‐forming functionalities with potential capacity‐recovering/self‐healing characteristics. Thus, on the basis of this exemplary work, structurally related boron‐based additives with tailored reductive/oxidative potentials, appropriate decomposition pathways, and interphase chemistries may represent a promising concept for electrolyte additive design.

## 4 Experimental Section

### 4.1 Additive Synthesis

*N*‐hydroxybenzamide (2.06 g, 15.0 mmol, 1.00 equiv) and phenylboronic acid (1.92 g, 15.8 mmol, 1.05 equiv) were added to a round‐bottom flask equipped with a stir bar and a Dean–Stark apparatus. Toluene (0.25 M, 60 mL) was added, followed by molecular sieves (4 Å, 7.5 g). The reaction mixture was stirred under reflux conditions for 16 h. After the reaction mixture was cooled to room temperature, the molecular sieves were filtered off, and the solvent was removed in vacuo. The crude product was purified by recrystallization from ethanol, yielding **2,5‐diphenyl‐1,3,4,2‐dioxazaborole (DOB)** as a white crystalline solid (2.16 g, 9.68 mmol, 65%).

### 4.2 Electrolyte Formulation

Electrolyte mixtures were prepared by mixing 1 M LiPF_6_ (battery grade; E‐Lyte Innovations GmbH) in an ethylene carbonate (EC) and ethyl methyl carbonate (EMC) solution mixture (3:7 by weight; battery grade; E‐Lyte Innovations GmbH). Electrolytes containing fluoroethylene carbonate (FEC; battery grade; BASF) and/or DOB were prepared by adding 0.189 mmol additive per gram of electrolyte (corresponding to 2 wt% FEC) to the previously mixed electrolytes. All electrolytes were prepared in an argon‐filled glovebox (MBraun) with H_2_O and O_2_ levels below 1 ppm.

### 4.3 Cell Setup

Wound multilayer NCM 523 || SiO_*x*_–graphite pouch cells (LiFun Technologies) balanced for a UCV of 4.3 V, with a nominal capacity of 200 mAh, were used for electrochemical investigations [10, 16]. The positive electrode has a mass loading of 16.5 mg cm^−2^, consisting of 94 wt% NCM 523 singlecrystalline powder, 4 wt% carbon black, and 2 wt% poly(vinylidene difluoride) binder (PVDF). The negative electrode has a mass loading of 8 mg cm^−2^, consisting of 94.8 wt% active material (90 wt% artificial graphite, 10 wt% SiO_*x*_)_,_ 1.4 wt% carbon black, 1.3 wt% carboxymethylcellulose (CMC), and styrene‐butadiene rubber (SBR). Prior to electrolyte filling, the cells were dried at 80 °C under reduced pressure (<0.05 mbar) for 12 h. Afterward, the cells were filled with 0.75 mL of electrolyte and sealed by a heat‐crimper at 165 °C for 5 s at a relative pressure of −80 kPa using a vacuum sealer (GN‐HS200V, Gelon Lib Group). For electrochemical investigation, a custom cell holder capable of applying 2 bar of pressure was used [16].

Overcharge experiments were carried out in a three‐electrode setup in T‐cells (Swagelok). A LiNi_0.5_Mn_1.5_O_4_ (LNMO; >99%; Sigma–Aldrich)‐based composite electrode (Ø12 mm) with an areal capacity of ≈0.36 mAh cm^−2^, made up of 90 wt% active material, 5 wt% carbon black (Super C65, Imerys Graphite & Carbon), and 5 wt% PVDF binder (Solef 5130, Solvay) coated on aluminum foil, was used as the working electrode. For the counter and reference electrodes, Ø12 and Ø8 mm lithium foil (500 μm; Gelon Lib Group) were used, respectively. The working and counter electrodes were separated by three layers of Ø12 mm polypropylene (PP) fiber separators (FS2190; Freudenberg) soaked in 120 μL of electrolyte, and the reference electrode was separated by two layers of the same separator soaked in 120 μL of electrolyte. Pouch cells were sealed, and T‐cells were assembled in a dry room atmosphere with a dew point of −50 °C or lower (0.16% relative humidity).

### 4.4 Electrochemical Investigation

Electrochemical investigation of NCM 523 || 10% SiO_*x*_–Gr pouch cells was performed using a Maccor 4000 battery testing system. After sealing, the cells were charged to 1 V and held at this voltage for 20 h to ensure adequate wetting. For SEI formation, the cells were charged to 3.5 V at a CC of 10 mA (≈0.05 C), held at a CV of 3.5 V for 1 h, and then discharged to 2.8 V with a CC of 10 mA (≈0.05 C). This cycle is considered cycle zero. The cells then underwent two charge/discharge cycles with upper cut‐off voltages (UCVs) of 4.3 V. For these two cycles, the cells underwent a CC charging step at 40 mA (≈0.2 C), followed by a CV step until the current drops to <4 mA (≈0.02 C), and finally a CC discharge step at 40 mA (≈0.2 C) to 2.8 V. After the formation cycles at 20 °C, the cells were subjected to either cycling test until reaching 70% SoH at 20 °C, 200 charge/discharge cycling at 20 °C, or high‐temperature storage experiments at 60 °C. For cycling tests, the cells were subjected to a CC charging step at 100 mA (≈0.5 C), followed by a CV step until the current drops to <4 mA (≈0.02 C) or 300 mAh (≈1.5 C) of capacity is reached, and finally a CC discharge step at 100 mA (≈0.5 C). At the last cycle, the cells were further discharged with a CV step at 2.8 V until the current dropped to <4 mA (≈0.02 C). For the storage experiment, the cells rested for 6 h at 60 °C after formation cycles, charged with the CC mode at 100 mA (≈0.5 C) to 4.3 V, followed by a CV step until the current drops to <4 mA (≈0.02 C). The cells were then rested for 720 h, and their OCV was measured. Finally, the cells were discharged with the CC mode at 100 mA (≈0.5 C) to 2.8 V. Afterward, the pouch cells were removed from the battery tester for gas volume measurement. Then, the pouch cells were reconnected to the battery tester at 60 °C and subjected to one charge/discharge cycle at 20 mA (≈0.1 C), followed by four cycles at 100 mA (≈0.5 C), with a CV step at the top of charge until the current dropped below 4 mA (≈0.02 C) to initiate the discharge. The 0.5 C cycles were used to determine the reversible discharge capacity after the storage experiments. At least two pouch cells per testing parameter were used to validate reproducibility.

Overcharge experiments were carried out at room temperature after letting the T‐cells rest for 3 h after cell assembly to allow for adequate electrolyte wetting. The cells were then charged with a CC mode (0.09 mA cm^−2^), and the potential of the working electrode was measured. Charging is terminated when 30 h of measurement time or 6 V is reached. At least two T‐cells per electrolyte mixture were used to validate reproducibility.

### 4.5 Gas Volume Measurements

The volume of gas formed inside the pouch cell was estimated by the Archimedes In Situ Gas Analyzer (AISGA) method [54]. The pouch cells were submerged in deionized water by hanging them from a balance (S256 Low Range—Force Sensors SMD3277‐010, 10 g, Strain Measurement Device) with a wire hook through a ≈1 mm hole at the sealed edge of the pouch cell. The DAQami v4.2.1 software (Measurement Computing MC, 10 Commerce Way, Norton, MA 02766, USA) was used to acquire data and generate signals.

### 4.6 SEM

SEM (Carl Zeiss AURIGA; Carl Zeiss Microscopy GmbH) was employed to examine the cross‐section and surface morphology of aged electrodes, respectively. EDX was performed at an accelerating voltage of 6 kV using an X‐MaxN 80 mm^2^ EDX detector (Oxford Instruments). INCA software was used to assess the spectra (Oxford Instruments). Cells were disassembled in a dry room atmosphere with a dew point of −50 °C or lower (0.16% relative humidity). For cross‐section SEM imaging, electrodes were cut using an ion beam cross‐section polisher (JEOL). Prior to the cross‐section cut and SEM analysis, 2 mL of dimethyl carbonate (DMC) was used to wash the electrode. To avoid moisture contact, samples were transferred using an air‐tight sample holder to the SEM analysis chamber.

### 4.7 ICP‐OES

Two 12 mm electrodes were extracted from two different cells for each electrolyte formulation. Electrode samples were digested in reversed aqua regia using a microwave system (Multiwave 7000, Anton Paar) prior to analysis by ICP‐OES. The measurements were performed on an ARCOS (Spectro Analytical Instruments GmbH) instrument, equipped with a Scott‐style spray chamber, a crossflow nebulizer, and an axially positioned plasma torch. The following emission lines were observed during analysis: Li (670.780 nm), Ni (221.648, 231.604, and 232.003 nm), Co (228.616, 237.862, and 238.892 nm), Mn (257.611, 259.373 nm), and Cu (224.700, 324.754, and 327.396 nm). The method and further parameters were adapted from Vortmann‐Westhoven et al. and Evertz et al. [55, 56].

### 4.8 ToF‐SIMS

Measurements were performed using an IONTOF ToF‐SIMS 5 instrument utilizing a 30 keV Bi nanoprobe in spectrometry (bunched) mode with a dual source column to obtain high‐mass resolution depth profiles. The primary ion gun was set to shoot Bi_3_^+^‐ions with an ion current of 2 pA over an area of 100 µm × 100 µm with a resolution of 128 × 128 pixels, and the analyzer was set to negative ion mode and a cycle time of 60 µs. For depth profiling, the Ar^+^ sputter beam was set to sputter 500 eV Ar^+^ ions with a current of 25 nA over an area of 300 µm × 300 µm in interlaced mode. Each sample was probed at three different locations to validate the results. The target current was measured before and after each measurement to compensate for fluctuations in the primary and sputter‐ion currents. To avoid contact with air, the samples were transferred to and introduced into the ToF‐SIMS device using an airtight sample carrier purchased from IONTOF. The samples were prepared for ToF‐SIMS analysis by washing the surface of the electrode five times with DMC to remove any excess electrolyte and conducting salt that may interfere with the measurement.

### 4.9 LIBS

Samples were analyzed using an ESLumen LIBS spectrometer (Elemental Scientific Lasers) combined with an imageGEO193 laser ablation system (Elemental Scientific Lasers). The system consists of a 193 nm ArF excimer laser with a two‐volume chamber (TwoVol3). A multi‐channel complementary metal–oxide semiconductor (CMOS) detector (spectrometer delay 0.1 µs, integration time 1 ms) and an isoplane spectrograph and intensified charge‐coupled device (ICCD) detector with a center wavelength of 695 nm, a gate delay of 0.1 µs, and intensifier gain of 1x were used simultaneously. The samples were analyzed with a helium gas flow of 1 L min^−1^, a laser fluence of 15.1 J cm^−2^, a repetition rate of 20 Hz, and a spot size of 100 µm. Data analysis was performed using the data visualization software, “iolite 4.9” [57]. This method has been previously reported [58].

### 4.10 DFT Calculations

Calculations were carried out within the framework of DFT [59, 60] as implemented in the ORCA program package [61, 62, 63]. Frontier molecular orbital energies were obtained using the 6‐311++G** Pople‐type basis set, which incorporates both double‐polarization functions to enhance basis set flexibility and diffuse functions to accurately describe species with extended electron densities, such as anions. The exchange–correlation potential was treated using the B3LYP functional [64, 65, 66]. Solvent effects were included via the conductor‐like polarizable continuum model (C‐PCM) [67], with ethylene carbonate (*ε* = 90.5) [68] as the implicit solvent. All structures were fully optimized prior to the subsequent energy evaluations.

## Disclaimer

The opinions and interpretations expressed in this Perspective are solely those of the authors and do not necessarily reflect the views of Small Science, its editors, or Wiley‐VCH GmbH.

## Conflicts of Interest

The authors declare no conflicts of interest.

## Supporting information

Supplementary Material

## Data Availability

The data that support the findings of this study are available from the corresponding author upon reasonable request.
