# Supplementary material for: The Exceptional Challenges of Si‐Based Anodes and the Eternal Hunt for Electrolyte Additives: A Perspective on the Example of a Novel Dioxazaborole‐Based Molecule
Source: Small Sci. 2026 Oct 22;6(11):e70429. doi: 10.1002/smsc.70429 (PMC13691293; doi:10.1002/smsc.70429)

## Supporting Information

# The Exceptional Challenges of Si-based Anodes and the Eternal Hunt for Electrolyte Additives – A Perspective on the Example of a Novel Dioxazaborole-based Molecule

*Anindityo Arifiadi,[1,2,#] Steffen Heuvel,[2,3,#] Lisa Balke,[2,4,#] Niklas M. Abke,[1] Alexandros Tsoufios,[1] Julius Buchmann,[1,2] Benjamin T. Manard,[5] C. Derrick Quarles Jr.,[6] Dominik Voigt,[1] Sascha Nowak,[1] Martin Winter,[1,2,7] Uwe Karst,[4] Frank Glorius,[2,3] * Johannes Kasnatscheew[1] **

*Dr. A. Arifiadi, N. M. Abke, A. Tsoufios, J. Buchmann, Dr. D. Voigt, Dr. S. Nowak, Prof. Dr. M. Winter, Dr. J. Kasnatscheew* *

[1] University of Münster, MEET Battery Research Center, Institute of Physical Chemistry

Corrensstr. 46, 48149 Münster, Germany

*E-mail: johannes.kasnatscheew@uni-muenster.de

*Dr. A. Arifiadi, S. Heuvel, Dr. L. Balke, J. Buchmann, Prof. Dr. M. Winter, Prof. Dr. F. Glorius*

[2] University of Münster, International Graduate School for Battery Chemistry, Characterization, Analysis, Recycling and Application (BACCARA)

Corrensstr. 40, 48149 Münster, Germany

*S. Heuvel, Prof. Dr. F. Glorius*

[3] University of Münster, Institute of Organic Chemistry

Corrensstr. 36, 48149 Münster, Germany

*E-mail: glorius@uni-muenster.de

*Dr. L. Balke, Prof. Dr. U. Karst*

[4] University of Münster, Institute of Inorganic and Analytical Chemistry

Corrensstr. 48, 48149 Münster, Germany

*Dr. B. T. Manard*

[5] Chemical Sciences Division, Oak Ridge National Laboratory

Oak Ridge, TN 37831, USA

*Dr. C. D. Quarles Jr.*
[6] Elemental Scientific, Inc.
Omaha, NE 68122, USA

*Prof. Dr. M. Winter*
[7] Helmholtz Institute Münster, IMD-4, Forschungszentrum Jülich GmbH
Corrensstr. 46, 48149 Münster, Germany

[#]*A. Arifiadi*, *S. Heuvel,* and *L. Balke* contributed equally to this work.

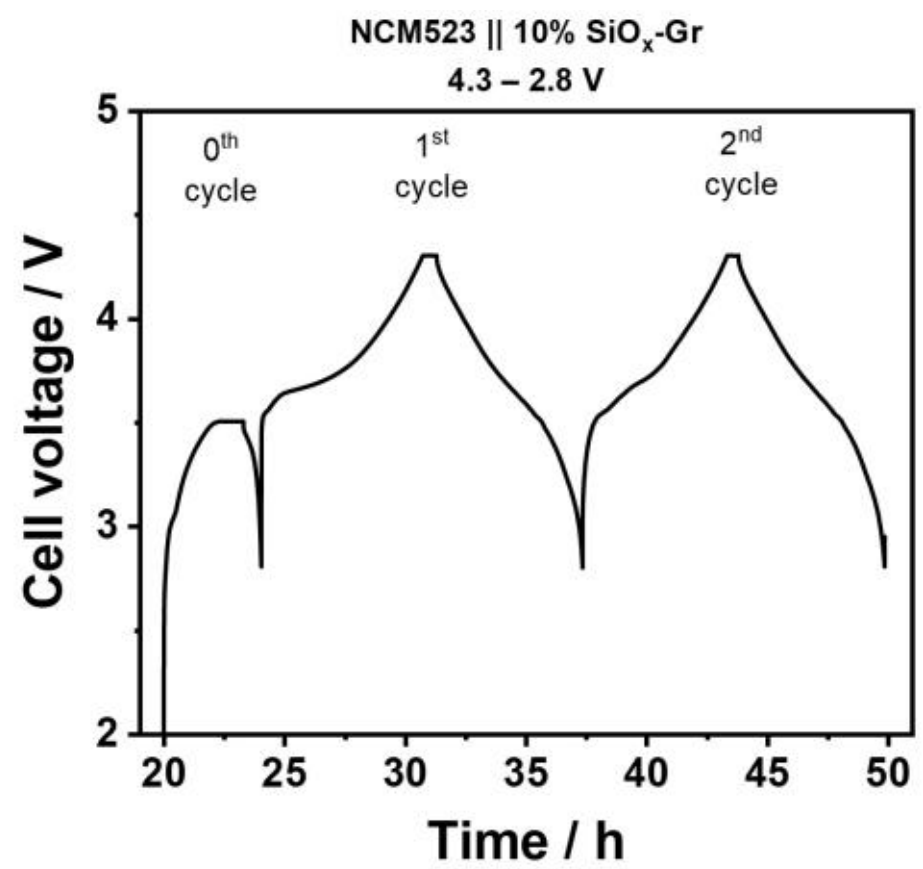


**Figure S1.** Cell voltage *vs.* time plot for a cell with the STD electrolyte during the formation cycles. The cell underwent a half-charge cycle to 3.5 V in the 0[th] cycle and two full-charge cycles in the 1[st] and 2[nd] cycles. Adapted from A. Arifiadi et al., *Small Sci.* **2026**, *6*, e202500637 (Copyright 2026, Wiley-VCH GmbH) under the Creative Commons Attribution License (CC BY 4.0).

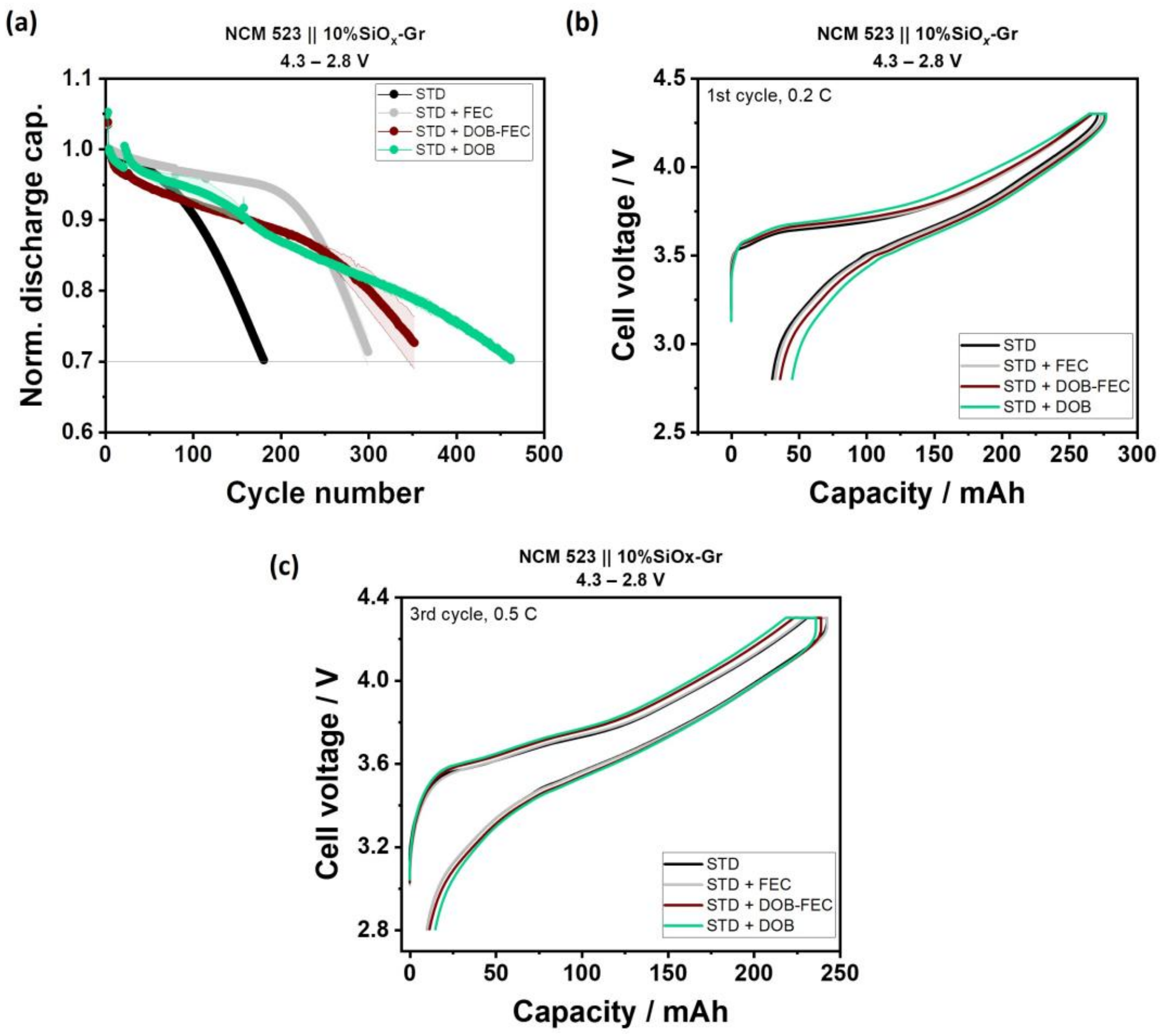


**Figure S2.** (a) Normalized discharge capacity *vs.* cycle number plot and voltage profile *vs.* capacity plots in the (b) first and (c) third cycle of cells with STD, STD + FEC, STD + DOB-FEC, STD + DOB electrolytes. Parts of the STD and STD + FEC data were adapted from A. Arifiadi et al., *Small Sci.* **2026**, *6*, e202500637 (Copyright 2026, Wiley-VCH GmbH) under the Creative Commons Attribution License (CC BY 4.0).

# 1. GENERAL SYNTHESIS EXPERIMENTAL

## 1.1. Glassware, Solvents and Reagents

All glassware and Teflon-coated magnetic stir bars were dried in an oven at 120 °C prior to use. All anhydrous solvents were commercially supplied and stored over 3 Å molecular sieves or dried using an activated alumina column drying system (toluene, $Et_2O$). Reagents were purchased from commercial sources and used as received.

## 1.2. Data Analysis

**NMR spectra** were recorded on Bruker Avance II 400, Agilent DD2 500 or DD2 600 spectrometers. All spectral data was acquired at 295 K. Deuterated solvents were purchased from Eurisotop ($CDCl_3$, deuteration > 99.8%; DMSO-$d_6$, deuteration > 99.8%). Chemical shifts (δ) are reported in parts per million (ppm) and referenced to $CDCl_3$ ($^1$H: 7.26 ppm; $^{13}$C: 77.16 ppm). Coupling constants (*J*) are given in Hertz (Hz) and refer to corresponding multiplicities (s = singlet, d = doublet, t = triplet, q = quartet, quin = quintet, hex = hextet, h = heptet, m = multiplet, app = apparent, br = broad signal, dd = doublet of doublets, etc.). The $^1$H NMR spectra are reported as follows: chemical shift (multiplicity, coupling constants, number of protons). **High resolution mass spectra (HRMS)** were recorded using electrospray ionisation (ESI) on a Bruker Daltonics, MicroToF spectrometer and calibrated using formate ion clusters.

## 1.3. Naming of Compounds

Compound names are those generated by ChemDraw Professional 23.1 software (PerkinElmer), following the IUPAC nomenclature.

# 2. SYNTHESIS EXPERIMENTAL DATA

## 2.1. N-Hydroxybenzamide

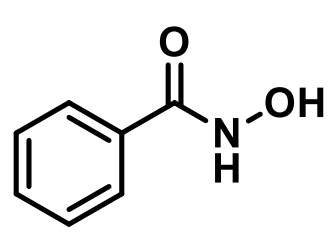

Hydroxylammonium chloride (2.8 g, 40 mmol, 1.0 equiv) was dissolved in $Et_2O$ (1 M, 40 mL) in a round-bottom flask. A $Na_2CO_3$ solution (4.2 g, 40 mmol, 1.0 equiv) was added and the reaction mixture was stirred at room temperature for 30 minutes. After the solution was cooled to 0 °C, benzoyl chloride (4.6 mL, 5.6 g, 40 mmol, 1.0 equiv) in $Et_2O$ (2 M, 20 mL) was added. The reaction mixture was stirred at room temperature for 16 hours. The solvent was removed in vacuo and the crude product was dissolved in EtOAc (200 mL). After the mixture was stirred under reflux conditions for 30 minutes, the hot solution was filtered and the solvent was consequently removed in vacuo. The product was recrystallized from EtOAc and washed with cold $Et_2O$ after filtration to yield *N*-Hydroxybenzamide as a white crystalline solid (3.50 g, 25.5 mmol, 64%).

**NMR Spectroscopy (*see spectra*):**

**$^1$H NMR** (400 MHz, DMSO-$d_6$): $\delta_H$ 11.21 (s, 1H), 9.03 (s, 1H), 7.79 – 7.71 (m, 2H), 7.56 – 7.48 (m, 1H), 7.48 – 7.41 (m, 2H) ppm;

**$^{13}$C NMR** (101 MHz, DMSO-$d_6$): $\delta_C$ 164.5, 132.9, 131.3, 128.5, 127.0 ppm.

**HRMS** (ESI–): m/z calculated for $C_7H_6NO_2$ $[M–H]^–$: 136.04040, found: 136.04028.

## 2.2. 2,5-Diphenyl-1,3,4,2-dioxazaborole (DOB)

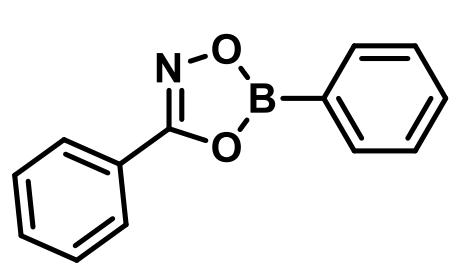

*N*-hydroxybenzamide (2.06 g, 15.0 mmol, 1.00 equiv) and phenylboronic acid (1.92 g, 15.8 mmol, 1.05 equiv; freshly recrystallized to avoid the corresponding boroxine) were added to a round-bottom flask equipped with a stir bar and a Dean–Stark apparatus. Toluene (0.25 M, 60 mL) was added, followed by molecular sieves (4 Å, 7.5 g). The reaction mixture was stirred under reflux conditions for 16 hours. After the reaction mixture was cooled to room temperature, the molecular sieves were filtered off, and the solvent was removed in vacuo. The crude product was purified by recrystallization from ethanol, yielding 2,5-diphenyl-1,3,4,2-dioxazaborole as a white crystalline solid (2.16 g, 9.68 mmol, 65%).

**NMR Spectroscopy (*see spectra*):**

**$^{1}$H NMR** (400 MHz, $CDCl_3$): $\delta_H$ 8.09 – 8.01 (m, 4H), 7.65 – 7.46 (m, 6H) ppm;

**$^{13}$C NMR** (101 MHz, $CDCl_3$): $\delta_C$ 165.1, 135.2, 132.8, 132.0, 129.0, 128.5, 127.0, 124.3 ppm;

**$^{11}$B NMR** (128 MHz, $CDCl_3$): $\delta_B$ 33.3 ppm.

**HRMS** (ESI–): m/z calculated for $C_{13}H_{10}BNO_2OH$ $[M+OH]^-$: 240.08375, found: 240.08359.

# 3. SYNTHESIS SPECTROSCOPIC DATA

**$^{1}$H NMR** (400 MHz, DMSO-$d_6$) of ***N*-Hydroxybenzamide (*see procedure*)**

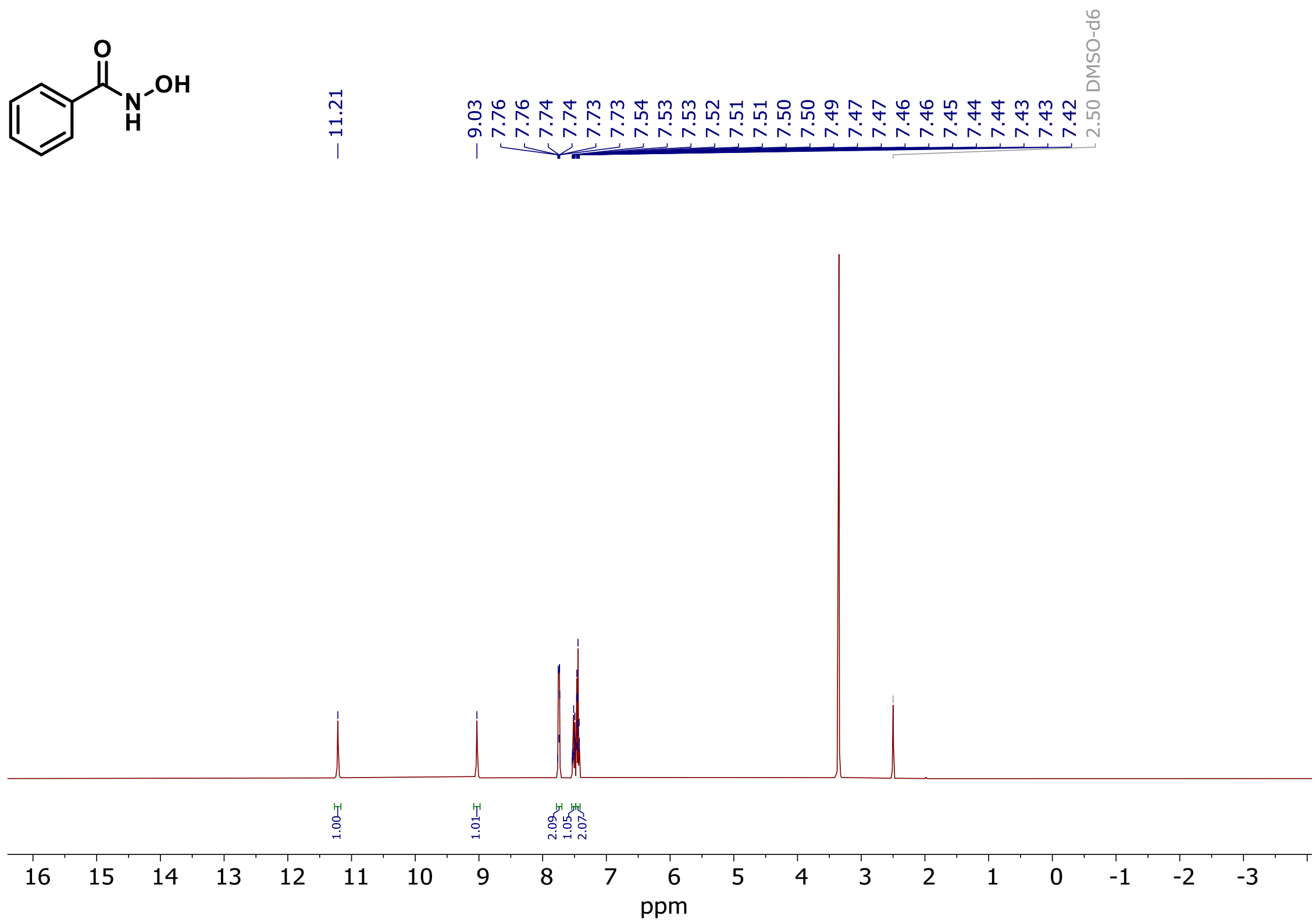

**$^{13}$C NMR** (101 MHz, DMSO-d$_6$) of ***N*-Hydroxybenzamide**

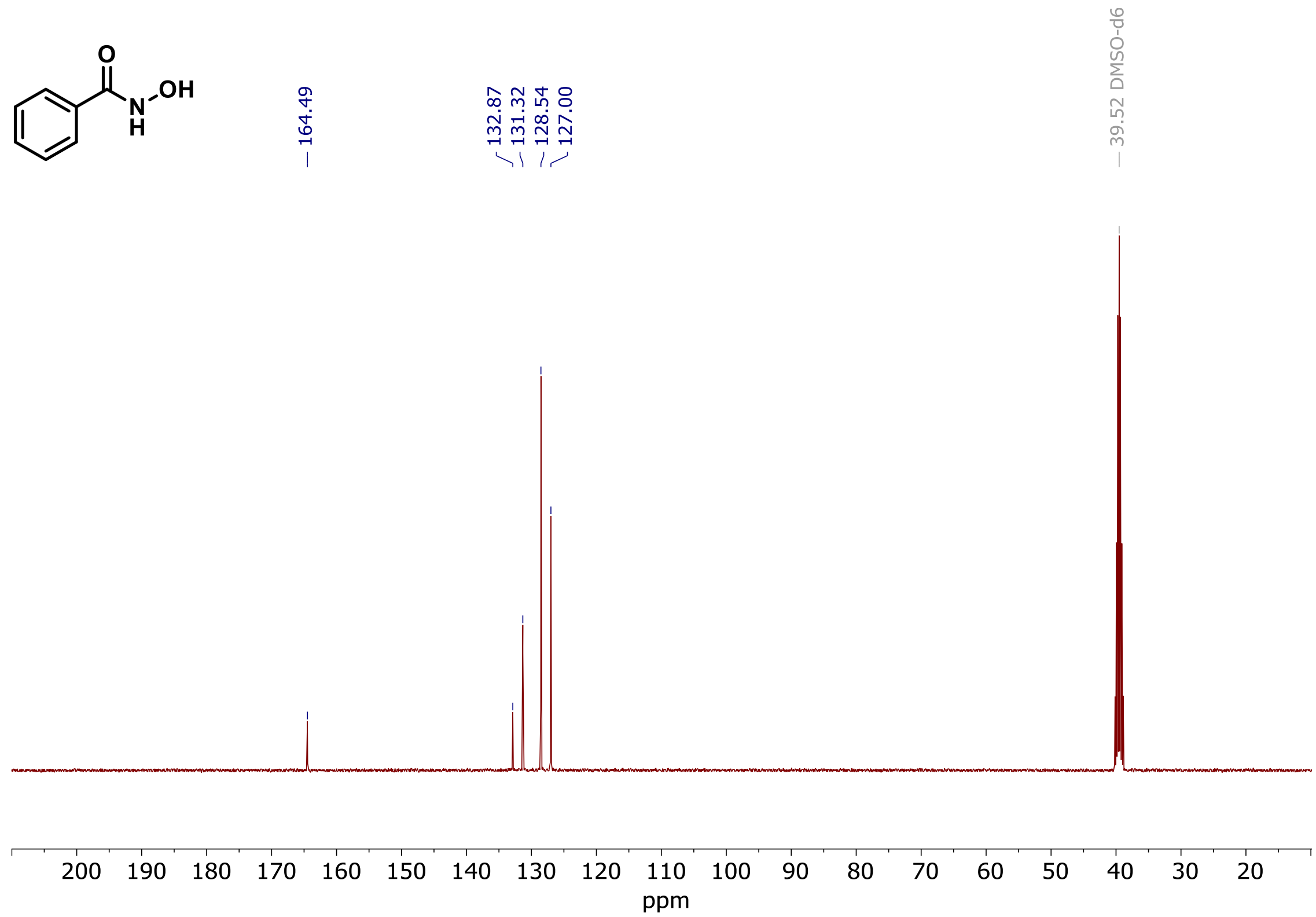


**$^{1}$H NMR** (400 MHz, CDCl$_3$) of **2,5-Diphenyl-1,3,4,2-dioxazaborole (DOB; *see procedure*)**

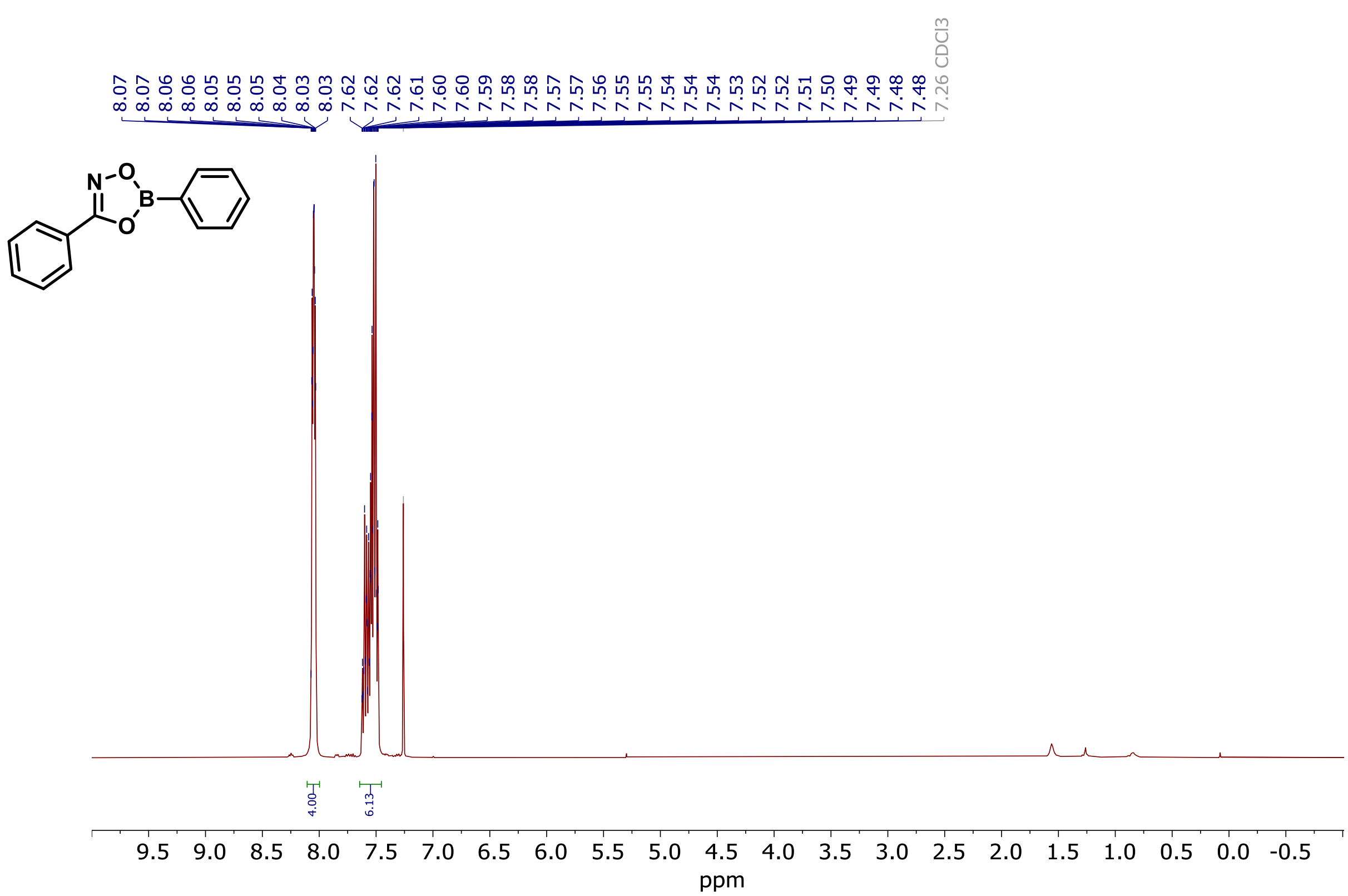

**$^{13}C$ NMR** (101 MHz, $CDCl_3$) of **2,5-Diphenyl-1,3,4,2-dioxazaborole (DOB)**

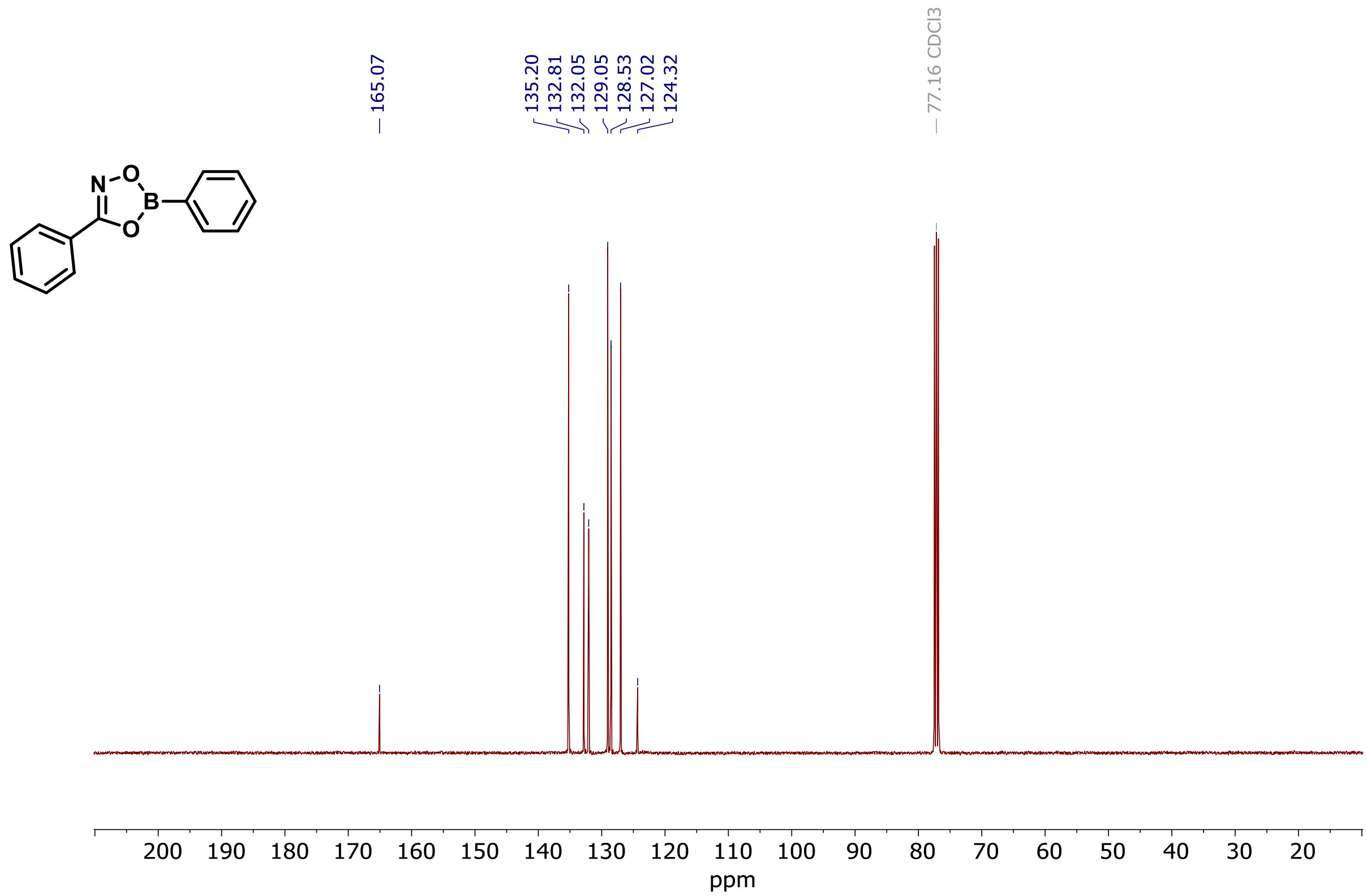


**$^{11}B$ NMR** (128 MHz, $CDCl_3$) of **2,5-Diphenyl-1,3,4,2-dioxazaborole (DOB)**

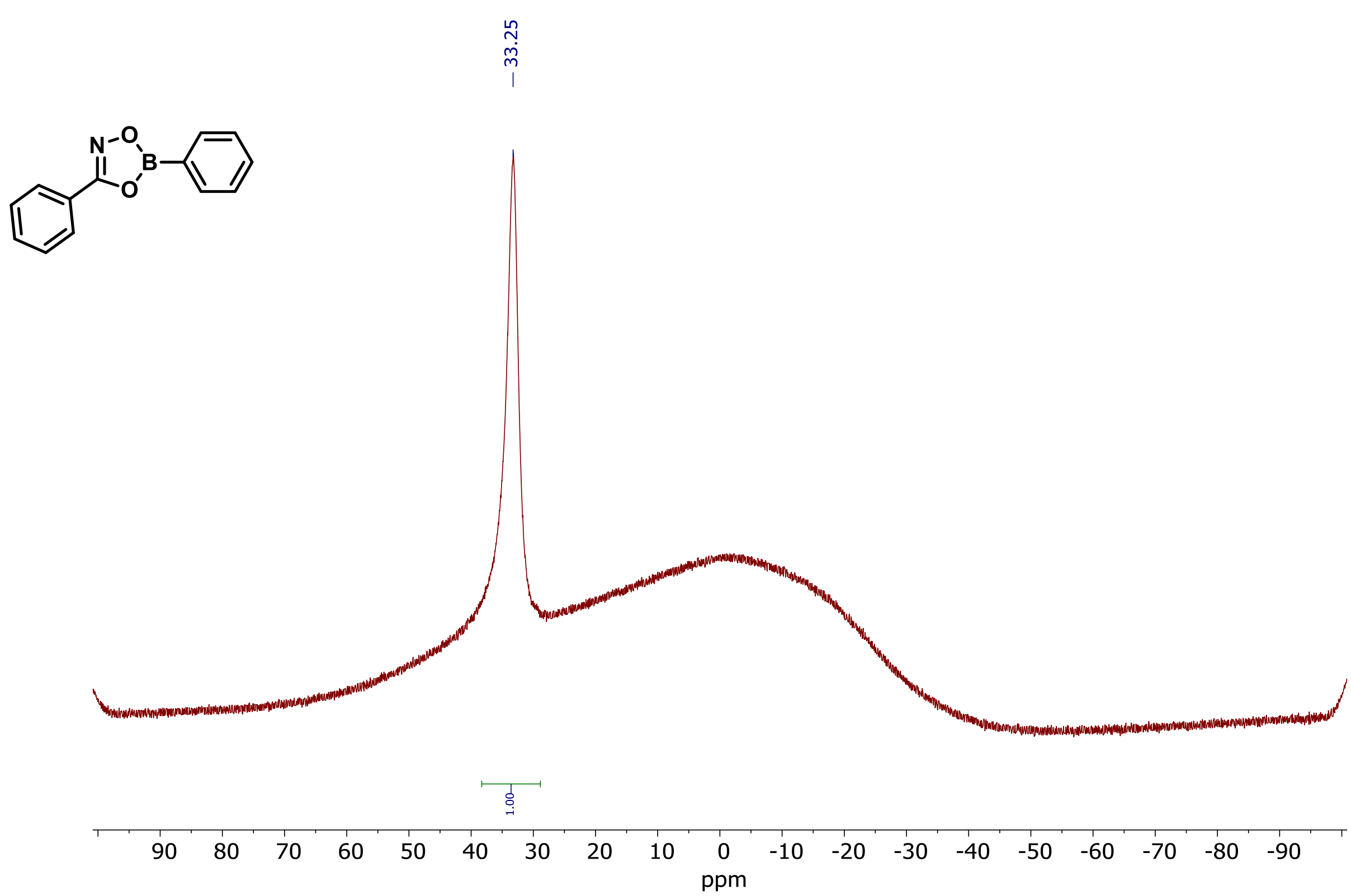

Supplement: Supplementary file 1 [file SMSC-6-e70429-s001.pdf]
